# GenomeFISH: genome-based fluorescence *in situ* hybridization for strain-level visualization of microbial communities

**DOI:** 10.1093/ismejo/wraf138

**Published:** 2025-07-07

**Authors:** J Pamela Engelberts, Jun Ye, Donovan H Parks, Eilish S McMaster, Allison S McInnes, Ben J Woodcroft, James G Volmer, Simon J McIlroy, Gene W Tyson

**Affiliations:** Centre for Microbiome Research, School of Biomedical Sciences, Queensland University of Technology, Translational Research Institute, Woolloongabba 4102, QLD, Australia; Australian Centre for Ecogenomics, School of Chemistry and Molecular Biosciences, The University of Queensland, Brisbane 4067, QLD, Australia; Australian Centre for Ecogenomics, School of Chemistry and Molecular Biosciences, The University of Queensland, Brisbane 4067, QLD, Australia; Centre for Microbiome Research, School of Biomedical Sciences, Queensland University of Technology, Translational Research Institute, Woolloongabba 4102, QLD, Australia; Centre for Microbiome Research, School of Biomedical Sciences, Queensland University of Technology, Translational Research Institute, Woolloongabba 4102, QLD, Australia; Centre for Microbiome Research, School of Biomedical Sciences, Queensland University of Technology, Translational Research Institute, Woolloongabba 4102, QLD, Australia; Centre for Microbiome Research, School of Biomedical Sciences, Queensland University of Technology, Translational Research Institute, Woolloongabba 4102, QLD, Australia; Centre for Microbiome Research, School of Biomedical Sciences, Queensland University of Technology, Translational Research Institute, Woolloongabba 4102, QLD, Australia; Centre for Microbiome Research, School of Biomedical Sciences, Queensland University of Technology, Translational Research Institute, Woolloongabba 4102, QLD, Australia

**Keywords:** fluorescence *in situ* hybridization (FISH), GenomeFISH, whole-genome hybridization, strains, single-cell genomics, high-throughput, single amplified genomes (SAGs), microbial ecology

## Abstract

Fluorescence *in situ* hybridization (FISH) is a powerful tool for visualizing the spatial organization of microbial communities. However, traditional FISH has several limitations, including limited phylogenetic resolution, difficulty visualizing certain lineages, and the design and optimization of new probes is time consuming and does not scale to the known diversity of microbial life. Here, we present GenomeFISH, a high-throughput, genome-based FISH approach that can differentiate strains within complex communities. Fluorescent probes are generated from the genomes of single cells, which are obtained from environmental or clinical samples through fluorescence activated single-cell sorting. GenomeFISH can distinguish between strains with up to 99% average nucleotide identity and was successfully applied to visualize strains in mock communities and human fecal samples. Given the superior sensitivity and specificity of GenomeFISH, we envisage it will become widely used for the visualization of complex microbial systems.

## Introduction

For over three decades, fluorescence *in situ* hybridization (FISH) has been an essential tool for visualizing microbial communities in a wide range of host-associated and environmental systems [1, 2]. FISH has been used to provide key insights into the spatial organization, morphology, and abundance of targeted microbial populations [3–5], and when combined with isotope labeling and staining approaches can be used to directly link the identity of microorganisms to their *in situ* function [6–8]. As such, FISH has given important context to genomic analyses and has been fundamental to advancing our understanding of microbial ecology.

Traditional FISH uses fluorescently labeled oligonucleotide probes that are designed to target conserved and variable regions of the 16S or 23S subunits of the ribosome, allowing the visualization of microorganisms at different phylogenetic levels. Ribosomal ribonucleic acid (rRNA)-based FISH has been central to the “full-cycle approach” to microbial ecology [9], where the community is first profiled based on ribosomal RNA gene sequencing, with probes designed to target rRNA sequences for populations of interest applied back to the original sample for visualization. However, rRNA-based FISH has several limitations that have prevented its application to certain environments and to the visualization of some microbial lineages. These include poor signal to background ratio due to high autofluorescence of the sample and/or low ribosome number of the target population, which typically occurs in slow growing microorganisms in oligotrophic environments [10]. Several alternative FISH techniques have been developed to address these fluorescent signal intensity limitations [11, 12]. The most widely adopted approach is CARD-FISH, where probes labeled with horseradish peroxidase (HRP) catalyze the deposition of fluorescently labeled tyramides in the target cell, increasing sensitivity up to 41 times over traditional FISH [13]. However, these FISH methods are labor intensive, requiring significant time to design, synthesize, and optimize new FISH probes, making it impractical to visualize even a small fraction of the diversity of microbial life discovered through metagenomics [14].

Existing FISH methods also have limited phylogenetic resolution due to the conserved nature of the ribosome, which only reliably allows visual differentiation at the genus level [15], potentially missing important species and strain information. One method that has been able to increase phylogenetic resolution for the visualization of microbial species is bacterial chromosomal painting (BCP) [16]. BCP targets the entire chromosome of the microorganisms of interest with probes generated from their genomic deoxyribonucleic acid (DNA). However, BCP has not been widely adopted, as the method is time-consuming, incompatible with traditional FISH, and requires isolates for the population of interest, excluding its use for the uncultivated majority of microorganisms. To overcome the existing limitations of FISH, new innovative approaches are required that increase labeled cell intensity, provide species and strain level resolution, and better scale to the diversity of microbial life.

Here, we present GenomeFISH, a genome-based FISH method that couples high-throughput, single-cell genomics with whole-genome hybridization to visualize microbial communities with strain-level specificity. GenomeFISH uses single cell genomes obtained from environmental and clinical samples, which are amplified, fragmented, labeled, and hybridized to visualize the target microorganism *in situ*. By targeting the whole genome, GenomeFISH overcomes the inherent limitations of traditional FISH, circumventing the need to design and optimize probes, while increasing sensitivity and specificity of target cell labeling. GenomeFISH was developed and optimized using pure cultures and applied to microbial communities in engineered and clinical systems to visualize species and strains of interest. GenomeFISH can be rapidly employed to any environmental sample to obtain fine-scale structural organization of the microbial community.

## Materials and methods

### Isolate growth conditions and fixation

GenomeFISH was first developed and optimized on a range of Gram-positive and Gram-negative in-house cultured isolates (Table S1). Of these isolates, *Escherichia coli* strains (UQ950, C43, EDL933, and CFT073), *Escherichia fergusonii*, *Klebsiella aerogenes*, *Pseudomonas aeruginosa*, and *Bacillus subtilis* were grown aerobically at 37°C in LB medium (1% tryptone, 1% NaCl, and 0.5% yeast extract). *Megamonas funiformis* and *Agathobacter rectalis* were cultured in anaerobic modified TY medium at 37°C.

Isolate cells were collected from 1 ml overnight cultures by centrifugation at 10000 g for 3 min and fixed overnight at 4°C in either 1% paraformaldehyde (PFA) (w/v) [17], for Gram-negative cells, or in 1X phosphate-buffered saline (PBS):50% ethanol (v/v), for Gram-positive cells. Samples were subsequently washed twice in 1X PBS and stored in 1X PBS:50% ethanol (v/v) at −20°C. Cells were fixed in 1% PFA instead of 4% PFA as this gave a brighter and more consistent signal from target cells indicating better permeability to GenomeFISH probes (Fig. S1). Aliquots of unfixed cells were also mixed in a 1:1 ratio with 50% glycerol (v/v) and stored at −80°C for single cell sorting.

### Environmental sample collection and fixation

GenomeFISH was also applied to two environmental samples: an established bioreactor performing nitrate-dependent anaerobic oxidation of methane [18] and two human fecal samples. Biomass was sampled anaerobically from the bioreactor. The fecal samples were collected at two time points (November 2022 and October 2023) from one healthy donor under the Queensland University of Technology ethics agreement (Approval Number 5203) and transferred into an anaerobic chamber within 15 min. Approximately 50 mg of sample was resuspended in 1000 μl of filter sterilized (Millex-GP 0.22 μm PES) anaerobic diluent and vortexed for 10 s before being filtered through a 35 μm FACS cell strainer (Falcon; 352 235) into a sterile 1.5 ml microfuge tube. Bioreactor and filtered fecal biomass was fixed overnight in 1% PFA (v/v) at 4°C. Filtered fecal biomass was also fixed overnight in 1X PBS:50% ethanol (v/v) for the visualization of Gram-positive cells. All fixed bioreactor and fecal samples were subsequently washed twice in 1X PBS and stored in 1X PBS:50% ethanol (v/v) at −20°C.

To preserve environmental samples for single cell sorting, unfixed biomass (bioreactor and human fecal samples) was mixed in a 1:1 ratio with 30% anaerobic glycerol (v/v) [19] and stored at −80°C. Prior to single cell sorting, human fecal samples were diluted 1:10 in 1000 μl of filter sterilized (Millex-GP 0.22 μm PES) anaerobic diluent.

### Deoxyribonucleic acid extraction and single cell sorting

For initial optimization purposes, GenomeFISH probes were generated from isolates by extracting DNA from 1 ml overnight cultures using the DNeasy PowerBiofilm Kit (Qiagen, Germany), following the manufacturer’s instructions. To generate GenomeFISH probes from single amplified genomes (SAGs) of isolates and microorganisms from environmental and clinical samples, single cells were sorted with FACS. Briefly, samples stored in glycerol were defrosted on ice, diluted 1:1000, stained with Syto 62, filtered through a 40 μm FACS cell strainer (Falcon; 352 235), and individually sorted into 96-well plates with a FACS Aria Fusion Flow Cytometer (BD Biosciences). The sort window was determined based on side scatter and red fluorescence (670/30 nm). To increase the chance of recovering SAGs from Patescibacteria in the bioreactor community, a second sort window was selected to collect smaller particles. Multiple displacement amplification (MDA) reactions were performed for each well using the REPLI-g Single Cell Kit (QIAGEN) as described previously [20], with the addition of a freeze–thaw cycle (10 min at −80°C and 10 min at 55°C) after the lysis of cells for 10 min at room temperature. MDA reactions were run on a QuantStudio 6 Flex Real-Time PCR System (Applied Biosystems). SAGs from wells with successful amplification were purified using the ZYMO DNA clean & concentrator kit (ZYMO Research), following the manufacturer’s instructions and DNA concentrations were measured with the Qubit dsDNA BR Assay Kit (Invitrogen).

To gain insight into the microbial community of the environmental samples and confirm that SAGs were retrieved from the dominant community members, total DNA was extracted from the biomass of the bioreactor and human fecal samples. For the bioreactor, DNA was extracted using the DNeasy PowerBiofilm Kit (Qiagen, Germany), following the manufacturer’s instructions. DNA was extracted from the homogenized, unfiltered human fecal samples at Microba Life Sciences (Brisbane) using the DNeasy 96 PowerSoil Pro QIAcube HT kit and the QIAcube HT DNA extraction system, following the manufacturer’s instructions.

### Metagenome assembled genome and single amplified genome recovery from isolates and environmental samples

DNA extracted from cultured isolates, from bioreactor and human fecal samples, and from SAGs was sent for sequencing to Microba Life Sciences (Brisbane). Libraries were prepared using the Illumina DNA Prep Library Preparation Kit as per the manufacturer’s instructions, with unique dual indexes and PhiX spike in at 2%. Samples were sequenced on the NovaSeq6000 in 2 × 150 bp format. DNA of isolates and SAGs were sequenced to 0.5 Gbp and bioreactor and human fecal samples were sequenced to 5 Gbp.

Reads from isolates and environmental samples were quality filtered, assembled, binned (bioreactor and fecal samples), and annotated using Aviary v0.6.0 [21]. In short, reads were assembled using metaSPAdes v3.15.4 [22] and binned with the binning algorithms MetaBAT v1 [23], MetaBAT v2 [24], MaxBin v2 [25], CONCOCT [26], VAMB [27], SemiBin v2 [28], and Rosella [29]. The best representative metagenome assembled genome (MAG) produced from the different binning algorithms was selected using DASTool v1.1.2 [30]. CheckM v2 [31] was used to determine completeness and contamination of each MAG; with only those over 50% complete with less than 10% contamination (medium to high quality MAGs) [32] retained for downstream analyses. Taxonomy was assigned to the MAGs with GTDB-Tk v2 [33]. To calculate the relative abundance of each MAG, reads were mapped to the MAGs using CoverM v0.6.1 [34] with the “genome” command. The average nucleotide identity (ANI) between isolate genomes, MAGs, and SAGs was calculated using FastANI v1.32 [35] and skani v0.2.1 [36]. Details of the isolate genomes can be found in Table S1. Details of the MAGs retrieved from the bioreactor and human fecal samples can be found in Tables S3 and S4, respectively.

Reads from each SAG were quality filtered and their genome coverage calculated by mapping the reads back to the MAGs of the corresponding isolate or environmental sample using CoverM v0.6.1 [34] with the “genome” command. Details of the coverage of each of the SAGs retrieved from the isolates, bioreactor sample, and fecal samples can be found in Tables S1, S3, and  S4, respectively.

### Deoxyribonucleic acid shearing and labeling for GenomeFISH probe generation

To generate GenomeFISH probes from isolates and SAGs, 2 μg of DNA was suspended in 130 μl of TE buffer in a Covaris AFA 6 × 16 mm microtube (Covaris) and sheared to ~130 bp fragments in a Covaris ME220 Focused-ultrasonicator (Covaris) using the following settings: temperature = 12°C, duration = 10 s, peak power = 75, duty factor = 25%, cycles per burst = 100, repeats = 30. DNA fragment size was determined using the QIAxcel Advanced System (Qiagen), following the manufacturer’s instructions. To pellet ~1 μg of DNA, 1/10^th^ volume of 3 M sodium acetate (pH 5.2) and two volumes of absolute ethanol was added. DNA was subsequently stored at −80°C for 30 min to overnight and centrifuged for 15 min at 12000 g. The pellet was washed twice in 80% ethanol, centrifuged for 10 min at 12000 g and dried in a SPD111V SpeedVac System (ThermoFisher Scientific). The pelleted DNA was chemically labeled with a selected fluorophore (Alexa546, Alexa594, or Alexa647) using the ULYSIS Nucleic Acid Labeling Kit (Invitrogen), following the manufacturer’s instructions, with the exception that 18 μl labeling buffer and 7 μl ULYSIS labeling reagent stock solution was used per reaction. The labeling reaction was further run for 30 min at 80°C instead of 15 min. Labeled probes were purified through a MicroSpin G-50 Column (Cytiva), with an initial centrifugation of 1 min at 750 g to resuspend the buffer, followed by 2 min at 1000 g to purify the probe. Probe concentration and labeling efficiency were determined using a PHERAstar *FS* (BMG Labtech, Note S1). Purified probes were diluted to 25 ng μl^−1^ in Tris-EDTA (TE) buffer and stored at −20°C. As a negative control for GenomeFISH experiments, calf thymus DNA (provided in the ULYSIS Nucleic Acid Labeling Kit) was sheared and labeled using the same protocol as described above.

### GenomeFISH

GenomeFISH was performed on fixed samples, following the direct-geneFISH protocol [15] with modifications to allow for whole-genome hybridization. Fixed biomass was applied to polytetrafluoroethylene (PTFE) printed slides and dried at 37°C for 10 min. Cells were dehydrated in an ethanol series (50%, 80%, 100%; 3 min each) and permeabilized with lysozyme (10 mg ml^−1^ lysozyme in 1X PBS, 0.05 M EDTA, and 0.1 M Tris–HCl, pH 8), with an incubation of 30 min at room temperature (for Gram-negative cells) or 1 h at 37°C (for Gram-positive cells). Slides were washed in MilliQ and 100% ethanol, further permeabilized with hydrochloric acid (0.2 M, 15 min at room temperature). The hydrochloric acid wash was extended to 1 h at room temperature to permeabilize archaea in the bioreactor sample. To image members of the *Patescibacteria* phylum, cells were dehydrated in an ethanol series (50%, 80%, 100%, 3 min each), permeabilized with lysozyme (10 mg ml^−1^, 1 h at 37°C) and achromopeptidase (60 U, 1 mM NaCl, 1 mM Tris–HCl, 30 min, room temperature), followed by a hydrochloric acid wash (0.2 M, 15 min at room temperature).

To each well, 10 μl of hybridization buffer was added (35% deionized formamide, 5X SSC (750 mM NaCl, 0.075 mM sodium citrate, pH 7), 20% dextran sulfate, 20 mM EDTA, 0.25 mg ml^−1^ sheared salmon sperm DNA, 0.25 mg ml^−1^ yeast RNA, 1X Denhardt’s blocking solution, and 0.1% SDS), together with 1 μl of genome-targeted probe (to a final concentration of 2.5 ng μl^−1^). As detailed in the direct-geneFISH protocol [37], initial experiments were performed using 35% formamide, after which formamide concentrations were further optimized (see Results). To allow GenomeFISH to be compatible with traditional FISH methods, all other hybridization conditions were kept the same as per the direct-geneFISH protocol [37]. Slides were incubated for 40 min at 85°C to denature the target DNA, followed by hybridization overnight at 46°C. After hybridization, slides were submerged in pre-warmed wash buffer (80 mM NaCl, 100 mM Tris–HCl pH 8, 5 mM EDTA, and 0.001% SDS) for 15 min at 48°C, followed by a 20 min wash at room temperature in 1X PBS, 1 min in MilliQ, and 2 min in 100% ethanol. Cells were counterstained with DAPI (1 μg ml^−1^) for 15 min at room temperature and washed in MilliQ and 100% ethanol. Slides were mounted with VECTASHIELD PLUS antifade mounting medium (VECTOR Laboratories) and microscopic analysis was performed with a Stellaris5 (Leica) laser scanning confocal microscope.

### 16S ribosomal ribonucleic acid–based fluorescence *in situ* hybridization

GenomeFISH can be combined with traditional 16S and 23S rRNA FISH by adding rRNA targeting probes during hybridization. For a comparison of signal strength between GenomeFISH and traditional FISH, both were applied to isolates and bioreactor samples. Traditional FISH was performed as previously described [9] with the following probes: Gam42a, targeting members of the *Gammaproteobacteria* [38], EUB-I, targeting bacteria in general [39], Amx.820, targeting most members of the family *Brocadiaceae* [40], and Ignav-b7–831, targeting members of the order *Ignavibacteriales* [3] (Table S2).

### Confocal microscopy, image processing, and data analysis

**Figure 1:**
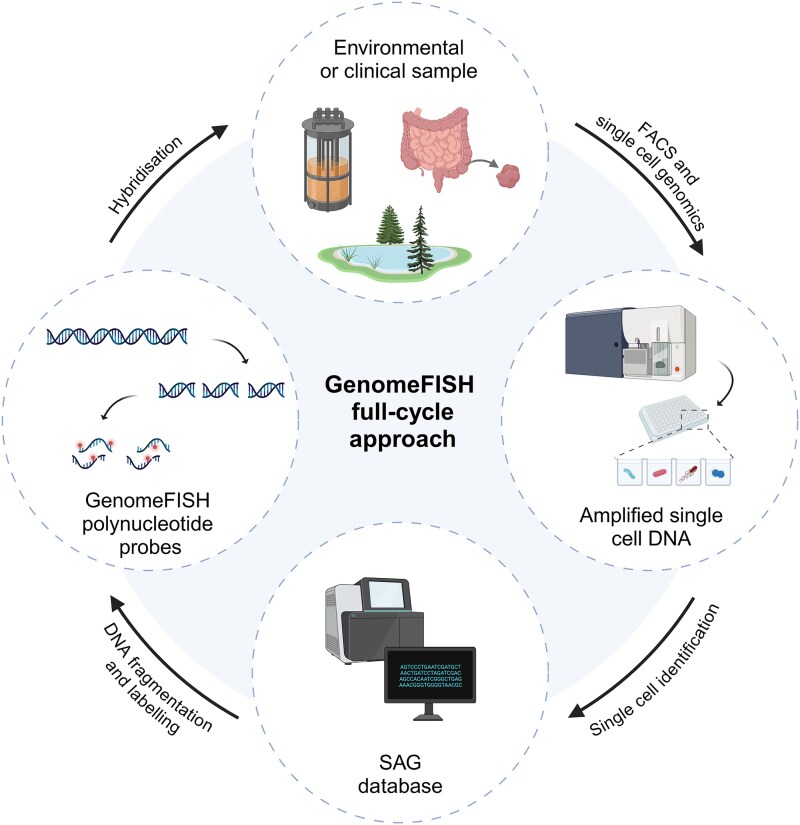
Schematic of the GenomeFISH-based full-cycle approach. Single cells are isolated from an environmental or clinical sample using FACS and are subjected to MDA to obtain sufficient DNA for probe generation. MDA products of SAGs of interest are fragmented to ~130 bp and fluorescently labeled to generate GenomeFISH polynucleotide probes. These probes are hybridized to the original sample for the visualization of microbial communities with strain-level specificity.

Composite images were generated with Leica Application Suite X (LAS X) v3.5.5 and further analysed using CellProfiler v4.2.5 [41] to generate signal intensity profiles of single cells. In short, all channels were aligned using the “mutual information” method. For images of cells from isolate cultures and the bioreactor community, primary objects were identified in the DAPI channel by thresholding with the Minimum Cross-Entropy method [42], with a Threshold correction factor of 1.3 and a Threshold smoothing scale of 1.5. The average signal intensities were subsequently measured for each of the objects (i.e. single cells) in each channel. The increase in signal intensities of GenomeFISH compared to traditional FISH was further calculated and confirmed with the Fiji software [43], using only non-saturated images. To measure signal intensities of *A. rectalis* strains in human fecal samples, GenomeFISH images were manually segmented with Fiji. Statistical analyses were performed in R v4.1.2 [44] using the package FSA [45]. *P* values were calculated using the Mann–Whitney *U*-test or Kruskal–Wallis test with post-hoc Dunn test. To increase resolution, linear deconvolution was applied to channels using the Diffraction PSF 3D FIJI plugin based on theoretical point-spread functions. Graphs were created in R v4.1.2 [44] using ggplot2 v3.3.5 and refined in Inkscape v1.2 (https://inkscape.org/). Figure 1 was created in BioRender. Cmr, C. (2024) https://BioRender.com/t97t048.

## Results

### Overview of GenomeFISH

GenomeFISH integrates high-throughput single cell genomics with whole genome-based hybridization with fluorescently labeled probes (Fig. 1). Single cells are obtained from environmental and clinical samples using fluorescence-activated cell sorting (FACS) and their genomic DNA amplified by MDA to obtain sufficient DNA for probe generation. Single cell MDA products are sequenced to identify microorganisms of interest, sheared to ~130 bp fragments, and fluorescently labeled (Note S1). These fluorescent probes are hybridized to fixed biomass from the original community to visualize target microorganisms with fluorescence microscopy.

### Development and optimization of GenomeFISH on pure cultures

**Figure 2:**
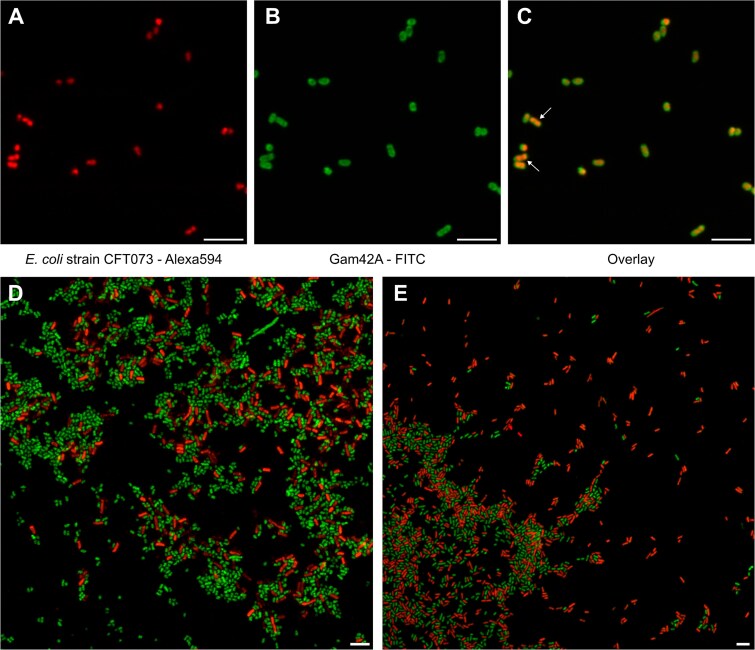
GenomeFISH and traditional FISH micrographs of isolates. (A–C) GenomeFISH and 16S rRNA-based FISH micrographs showing the same field of view of *E. coli* strain CFT073. Isolate cells were hybridized with GenomeFISH probes generated from *E. coli* strain CFT073 (A) and the Gam42a FISH probe targeting the 23S rRNA [38] (B). Panel C shows an overlay of the GenomeFISH and FISH micrographs. White arrows indicate cells that appear to be starting to divide. (D) GenomeFISH applied to a mock community, consisting of *E. coli* strain EDL933 and *B. subtilis*, using GenomeFISH probes for *E. coli* EDL933 (Alexa546, green) and *B. subtilis* (Alexa594, red). (E) GenomeFISH applied to a mock community, consisting of *E. coli* strain CFT073 and *P. aeruginosa* (red), using GenomeFISH probes for *E. coli* CFT073 (Alexa546, green) and *P. aeruginosa* (Alexa594, red). Scale bars are 5 μm.

GenomeFISH was developed and optimized using a range of Gram-positive and Gram-negative isolates, including *E. coli* strains (CFT073, EDL933, C43, and UQ950), *E. fergusonii*, *K. aerogenes*, *P. aeruginosa*, *M. funiformis*, *B. subtilis*, and *A. rectalis* (Table S1). GenomeFISH probes were generated from extracted genomic DNA and hybridized to fixed cells of each isolate (see Methods, Note S1, and Table S2), resulting in strong fluorescence for each cell, confirming probe penetration and hybridization to the genome (Figs 2A and S2). Fluorescent probes generated from calf thymus DNA did not label the cells, confirming the observed GenomeFISH signal was not due to non-specific probe binding (Fig. S3). To confirm the specificity of GenomeFISH for its target species, probes were generated for four isolates labeled with different fluorochromes (*E. coli* EDL933, *E. coli* CFT073, *B. subtilis*, and *P. aeruginosa*) and simultaneously applied to mock communities that consisted of different combinations of these species. GenomeFISH clearly distinguished each species within these mixtures (Fig. 2D and E). Overall, the application of GenomeFISH to diverse isolates showed that the method can be used for the targeted visualization of a range of microorganisms.

Unlike traditional FISH where the fluorescence signal is typically uniform across the cell (i.e. based on the distribution of the ribosomes), the GenomeFISH signal was more localized, consistent with targeting the genome (Fig. 2A–C). Given this signal localization, it was possible to visualize genome duplication prior to cell division in *E. coli* strain CFT073 during exponential growth (Fig. 2C). Importantly, GenomeFISH increased signal intensity by up to 3 times compared to traditional FISH (*P* < .001, Fig. S4), demonstrating that GenomeFISH outperforms traditional FISH even in cells that contain a high number of ribosome binding sites (up to 72 000 ribosomes) [46]. This suggests that GenomeFISH will substantially outperform traditional FISH in environmental samples, where microorganisms typically have a lower number of ribosomes.

### Strain differentiation in mock communities

**Figure 3:**
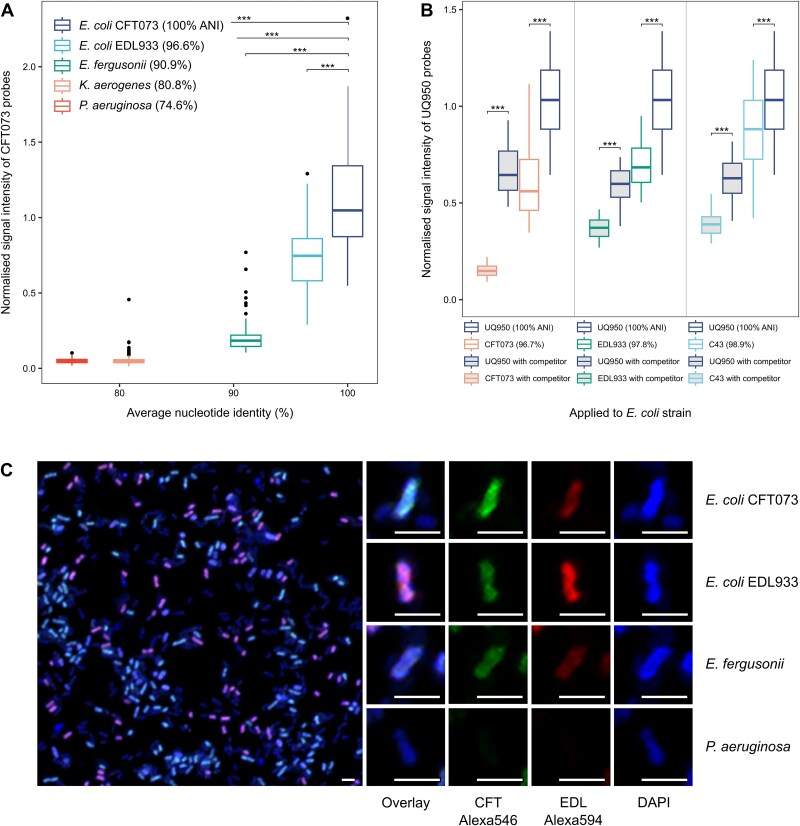
Validation of the ability of GenomeFISH to identify strains. (A) Relationship between normalized GenomeFISH signal intensity (GenomeFISH/DAPI signal intensity) and ANI when GenomeFISH probes generated from *E. coli* strain CFT073 were hybridized individually to microorganisms with ANI values between 74.6% and 96.6%. For each microorganism, signal intensities were measured for three fields of view. (B) Relationship between normalized GenomeFISH signal intensity (GenomeFISH/DAPI signal intensity) and ANI when GenomeFISH probes generated from *E. coli* strain UQ950 were hybridized individually to *E. coli* strains C43, EDL933, and CFT073, either with or without a competitor probe (unlabeled GenomeFISH probes generated from the respective non-target strain). For each microorganism, signal intensities were measured for five fields of view. (C) GenomeFISH applied to a four-member mock community, consisting of *E. coli* CFT073 and EDL933, *E. fergusonii*, and *P. aeruginosa*, using GenomeFISH probes for *E. coli* CFT073 (Alexa546, green) and *E. coli* EDL933 (Alexa594, red). *E. coli* CFT073 cells appear cyan, *E. coli* EDL933 cells appear magenta, and *E. fergusonii* cells appear light blue. No hybridization occurred for *P. aeruginosa*. Scale bars are 1 μm. Significance: *P* < .05 (^*^), *P* < .01 (^**^), and *P* < .001 (^***^).

To test the specificity of GenomeFISH, probes generated from *E. coli* strain CFT073 were hybridized to isolates with ANI values ranging from 74.6% (order-level) to 96.6% (strain-level [35], Figs 3A and S5). As the orientation of cells in the image focal plane can influence signal intensities (Fig. S6A), the GenomeFISH signal was normalized to a DNA counterstain (DAPI). These two signals were directly correlated (*R*^2^ = 0.90, *P* < .001, Fig. S6B) and remained consistent across all growth phases of *E. coli* (Fig. S6C and D, Note S2). This initial comparison of normalized signal intensities demonstrated that GenomeFISH could differentiate between strains with up to 96.6% ANI (*P* < .001, Figs 3A and S5).

To assess if GenomeFISH could distinguish between more closely related strains (>96.6% ANI), probes generated from *E. coli* strain UQ950 were hybridized to *E. coli* strains C43 (98.9% ANI), EDL933 (97.8%), and CFT073 (96.7%). GenomeFISH could clearly distinguish between strains with up to 97.8% ANI (*P* < .001, Fig. 3B). To enhance the phylogenetic resolution of GenomeFISH, “competitor probes” [38] were assessed for their ability to reduce non-target probe binding. Competitor probes are unlabeled probes that preferentially bind to the non-target genome sequence, blocking imperfectly matched labeled probes. Labeled GenomeFISH probes prepared from *E. coli* UQ950 were applied to *E. coli* strains (UQ950, C43, EDL933, and CFT073) with and without the inclusion of competitor probes (Fig. 3B). With the inclusion of competitor probes, GenomeFISH can differentiate between strains with up to 98.9% ANI (*P* < .001), demonstrating superior phylogenetic resolution to existing FISH methods.

The ability of GenomeFISH to differentiate strains and species was subsequently tested in a mock community. Probes targeting *E. coli* strains CFT073 (Alexa546) and EDL933 (Alexa594), acting as competitor probes for one another, were simultaneously applied to a mixture of isolates, consisting of *E. coli* CFT073, *E. coli* EDL933, *E. fergusonii*, and *P. aeruginosa*. To confirm that GenomeFISH differentiated between each member of the mock community, signal intensities (for Alexa546 and Alexa594) were measured and plotted for each microbial cell. Visual observation and measured signal intensities confirmed clear separation of the four populations (Figs 3C and S7).

### Optimization of signal specificity and intensity

**Figure 4:**
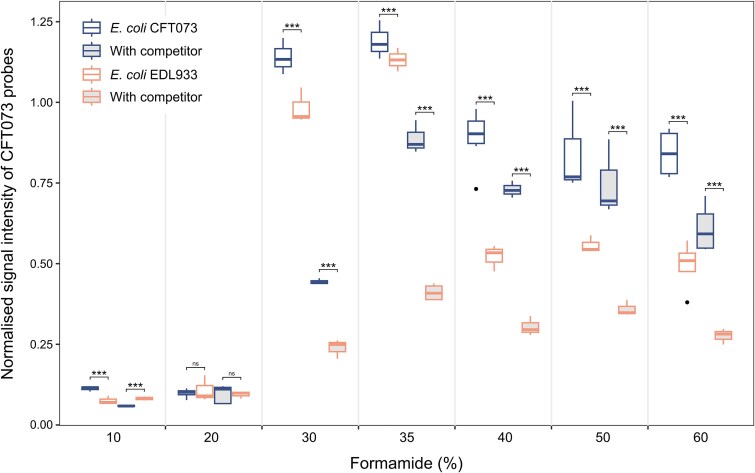
The effect of formamide concentration on the ability of GenomeFISH to differentiate strains. Formamide dissociation curves for the hybridization of GenomeFISH probes generated from *E. coli* strain CFT073 to *E. coli* CFT073 and *E. coli* EDL933, either with or without a competitor probe (unlabeled GenomeFISH probes generated from the respective non-target strain). Signal intensities were measured for five fields of view. Significance: *P* < .05 (^*^), *P* < .01 (^**^), *P* < .001 (^***^), not significant (ns).

For existing FISH methods, the concentration of formamide in the hybridization buffer is used to determine probe binding stringency [11, 37]. To determine the influence of formamide (FA) concentration on GenomeFISH probe binding, FA dissociation curves were generated for *E. coli* CFT073 GenomeFISH probes applied to both the target species and a closely related strain (EDL933). No signal was observed with ≤20% FA, indicating the importance of FA in the denaturation of the genome to allow probe binding. Maximum signal intensity of GenomeFISH probes generated from *E. coli* CFT073 was 30–35% FA and optimal stringency was achieved at 40%, where the two strains could be clearly separated based on measured signal intensity (*P* < .001, Fig. 4). The addition of an unlabeled competitor probe targeting EDL933 substantially increased the specificity of the labeled GenomeFISH probe at all FA concentrations where signal was observed (>20% FA; Fig. 4). These results indicate that formamide optimization is not essential for GenomeFISH but can be used to achieve greater specificity.

As the binding of DNA probes is influenced by their GC content [37], formamide dissociation curves were generated for GenomeFISH probes for microbial species that covered the known range of GC for bacteria (32% to 66%, Fig. S8) [47]. Consistent with previous studies [37], the optimal FA% was positively correlated with GC content of the target strain (Fig. S8). However, while the optimal FA% varied with GC content, the signal intensity was consistently high at 35%–40% FA concentrations for all tested strains. Based on these results, we used 35% FA to visualize microorganisms in environmental and clinical samples (see below) and recommend this as an ideal starting FA% when applying GenomeFISH.

### Optimizing single cell genome amplification for generation of GenomeFISH probes

A major advancement of GenomeFISH is the ability to rapidly generate probes from SAGs directly sorted from microbial communities. To verify that SAGs can be used as GenomeFISH probes, individual cells from *E. coli* strains CFT073 and EDL933 were sorted into a 96-well plate using a fluorescence-activated cell sorter (FACS) and amplified using MDA. Sequences from successfully amplified SAGs were mapped to the reference genomes with coverage ranging from 22%–100% for CFT073 and 59%–100% for EDL933 (Table S1). GenomeFISH probes were generated from SAGs of strain CFT073 and hybridized to fixed cells (with 35% formamide), allowing successful visualization of *E. coli* strains with fluorescent signal intensity proportional to the coverage of the genome (*P* < .001, Fig. S9). Even probes with low genome coverage (as little as 22%) gave signal above background and could be used to differentiate between strains (*P* < .001, Fig. S9). The ability to use SAGs in GenomeFISH circumvents the need to culture microorganisms of interest and overcomes the labor-intensive steps of probe design and optimization required in other FISH methods [2, 11, 37], making the method high-throughput and scalable for application to natural microbial communities.

### Application of GenomeFISH to a low complexity bioreactor community

To show the ability of GenomeFISH to visualize bacterial and archaeal species in low complexity microbial communities, GenomeFISH was applied to a bioreactor performing anaerobic methane oxidation (AOM) coupled to nitrate reduction [18]. The community in the bioreactor was first characterized using metagenomics, leading to the recovery of 15 medium- to high-quality metagenome-assembled genomes (MAGs) that represented 83% of the community (Table S3). FACS-based single cell genomics led to the recovery of 20 SAGs (with >10% genome coverage, Table S3), representing 69% of the community, including the two most abundant populations in the system, “*Candidatus* Methanopredens nitroreducens” (58.6% relative abundance) and “*Candidatus* Kuenenia stuttgartiensis” (3.9%). SAGs were also recovered for *SURF-28 sp003599395,* a species belonging to the phylum *Bacteroidota* (2.9%), and “*Candidatus* Chazhemtobacterium aquaticus”, a species from the *Patescibacteria* (1.6%, Table S3).

GenomeFISH probes generated from the best quality SAGs (49% to 85% genome coverage) were hybridized to the original bioreactor community, revealing morphologically distinct populations (Fig. S10). These morphologies were consistent with traditional FISH observations of populations for which FISH probes exist [3]. GenomeFISH reached signal intensities up to 27× higher than traditional FISH (*P* < .001, Fig. S11). However, visualization was successful for “*Ca.* Kuenenia stuttgartiensis” using a SAG with a genome coverage of 12% (Fig. S12). GenomeFISH was also used to visualize the *Patescibacteria* “*Ca*. C. aquaticus” (Fig. S10), for which no traditional FISH signal was observed (from the EUB-III probe); presumably due to the reported low number of ribosomes for members of this phylum [48]. “*Ca*. C. aquaticus” appeared as small cocci with a diameter of ~0.5 um, which is within the size range of other members of the *Patescibacteria* phylum [49]. Together, these results highlight the high sensitivity of GenomeFISH and its potential to rapidly visualize novel microorganisms in environmental samples.

### Application of GenomeFISH to human fecal samples

**Figure 5:**
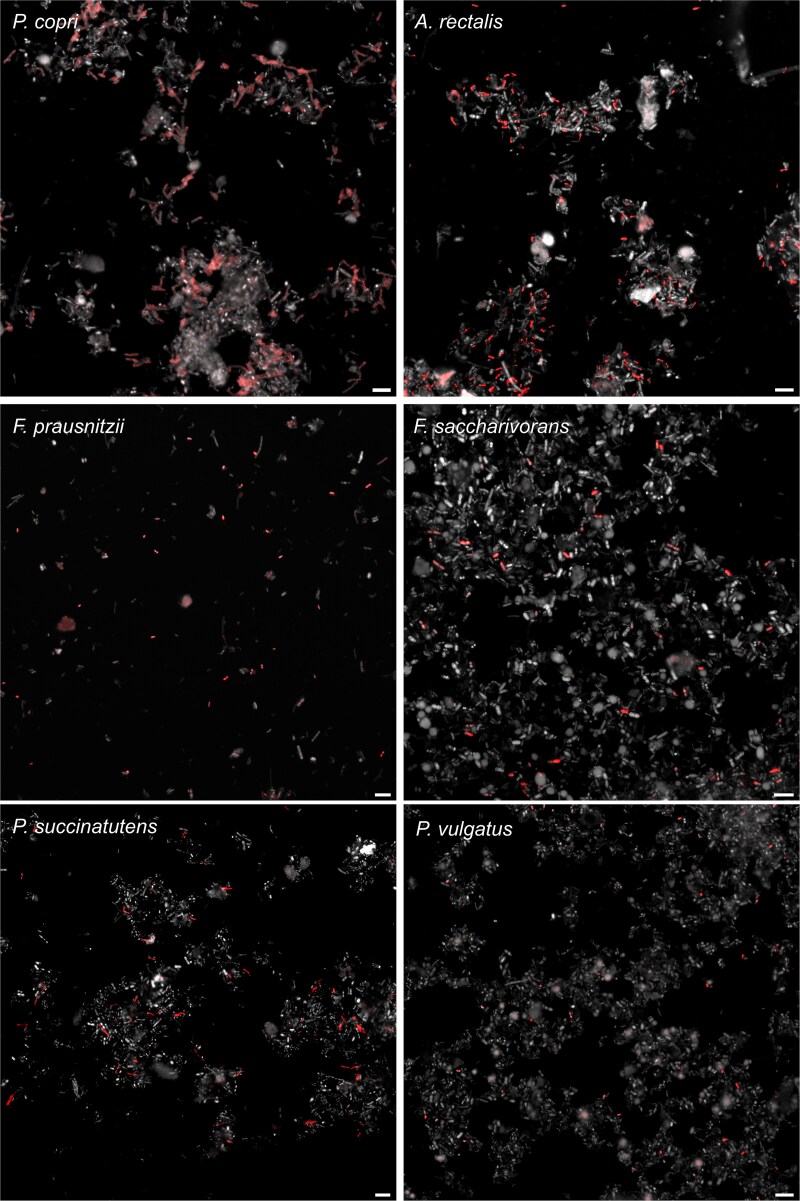
GenomeFISH micrographs of microbial populations in a human fecal sample. GenomeFISH micrographs show *Prevotella copri* (top left), *A. rectalis* (top right), *Faecalibacterium prausnitzii* (middle left), *Fusicatenibacter saccharivorans* (middle right), *Phascolarctobacterium_A succinatutens* (bottom left), and *Phocaeicola vulgatus* (bottom right) hybridized with their respective GenomeFISH probes. GenomeFISH signal appears red and DAPI signal gray. Scale bars are 5 μm.

**Figure 6:**
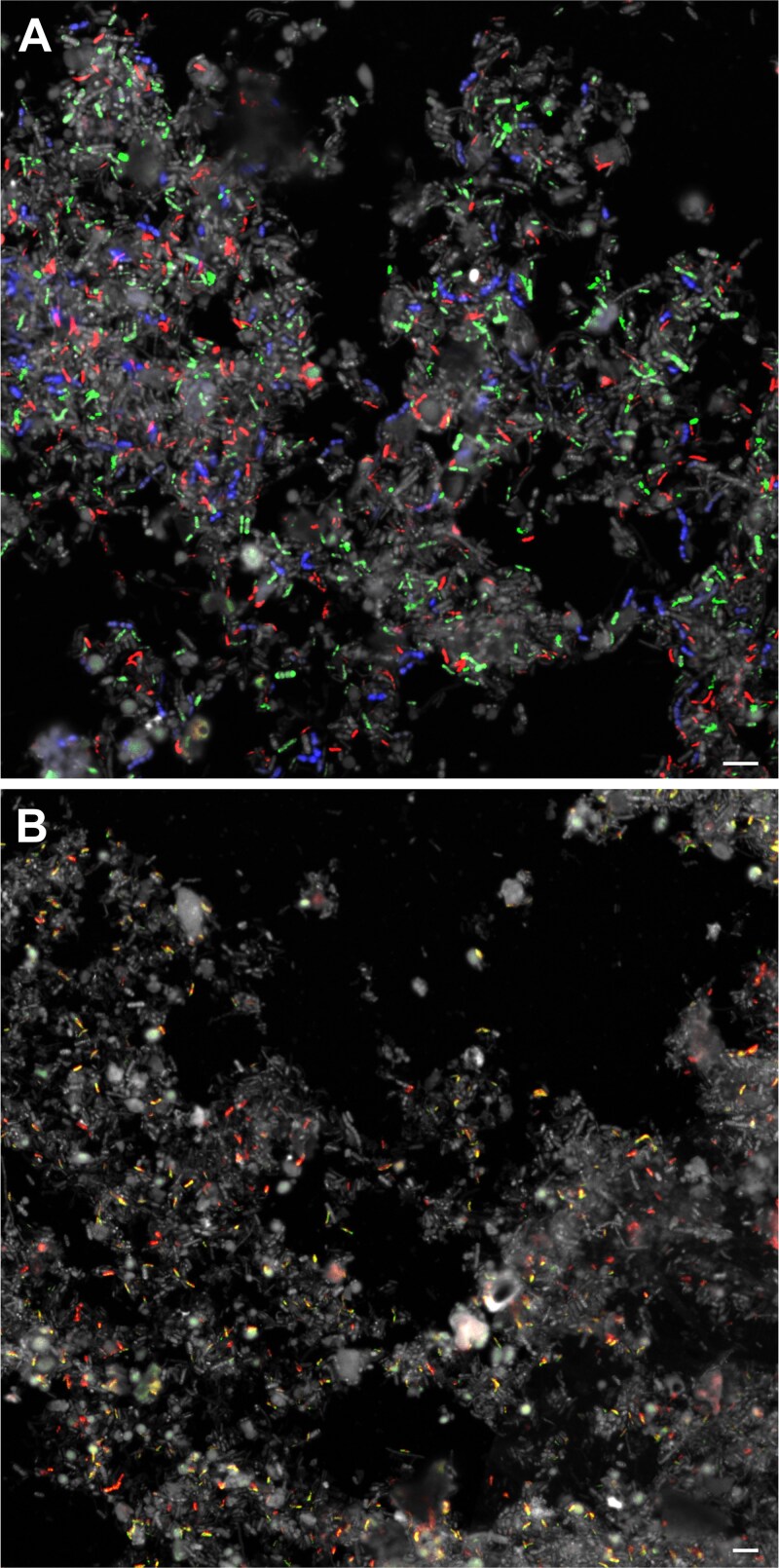
GenomeFISH micrographs of species and strains in human fecal samples. GenomeFISH micrographs showing human fecal samples hybridized with GenomeFISH probes generated from (A) *P. succinatutens* (green), *A. rectalis* (red), and *F. saccharivorans* (blue) or (B) two strains *of A. rectalis* (yellow and red). DAPI is shown in gray. Scale bars are 5 μm.

GenomeFISH was subsequently applied to a human fecal sample to demonstrate its ability to visualize microorganisms in a more complex microbial community. Metagenomics on the fecal sample led to the recovery of 41 medium- to high-quality MAGs and single cell genomics yielded 22 SAGs (>10% genome coverage) from 13 bacterial species (Table S4). GenomeFISH probes were generated for *Prevotella copri* (6.3% relative abundance), *A. rectalis* (3.8%), *Faecalibacterium prausnitzii* (1.4%), *Fusicatenibacter saccharivorans* (1.09%), *Phascolarctobacterium_A succinatutens* (0.8%), and *Phocaeicola vulgatus* (0.73%) and hybridized to the original fecal sample, allowing the visualization of each species (Figs 5 and S13). Using a combination of probes targeting *P. succinatutens*, *A. rectalis*, and *F. saccharivorans* in a single hybridization demonstrated the ability to simultaneously visualize and distinguish multiple species within a complex microbial community using GenomeFISH (Fig. 6A).

To demonstrate that GenomeFISH can reach strain-level resolution in natural microbial communities, SAGs were collected from a second sample from the same donor, which was dominated by a distinct *A. rectalis* strain (98% ANI, see Table S4 and Note S3). This enabled the generation of GenomeFISH probes for two *A. rectalis* strains, each unique to their respective fecal sample. Probes targeting the strains were hybridized to each sample, as well as to a homogenized mixture of the two samples. Visual assessment using GenomeFISH confirmed the presence of one *A. rectalis* strain in the individual samples and of two discrete strains in the mixed sample (Figs 6B and S14). These results confirm the ability to rapidly generate GenomeFISH probes to visualized natural microbial communities with superior sensitivity and specificity relative to traditional FISH approaches.

## Discussion

Here we present GenomeFISH, an innovative and high-throughput genome-based FISH method that can visualize natural microbial communities with strain-level resolution. We demonstrate the widespread applicability of GenomeFISH by visualizing microorganisms that span both bacterial and archaeal domains in mock communities, an engineered bioreactor, and clinical samples. We show that GenomeFISH has superior phylogenetic resolution to existing FISH methods, distinguishing between strains with up to 99% ANI. GenomeFISH also increased signal intensities by >27× compared to traditional FISH for tested species. Given the average genome will have >23 000 probe binding sites per genome copy (~3 Mb genome; 130 bp probes), we estimate GenomeFISH will consistently reach substantially higher signal intensities than standard rRNA-based FISH methods.

The superior phylogenetic resolution and sensitivity of GenomeFISH will provide unprecedented insights into the ecology of microbial communities that are missed when using existing FISH methods. For example, GenomeFISH allows the characterization of the spatial distribution and physical associations of co-existing species and strains, giving valuable insights into their respective niches and collective contribution to the ecology of the system. The increased sensitivity of the method will also enable the visualization of lineages otherwise missed due to low-ribosome numbers per cell and/or high autofluorescence of the environmental matrix they colonize i.e. soil, sediment, plants, and corals [50, 51]. GenomeFISH also allows the visualization of novel species as it bypasses the need to design probes based on ribosomal RNA sequences, which are often missing from MAGs [52].

GenomeFISH brings the “full-cycle approach” for microbial ecology into the meta-omic age, where our genomic analysis and visualization are both based on the genome. We also have the ability to better scale to the known diversity of microbial life, where hundreds of probes can be generated simultaneously and matched to the genomes of key populations. Populations of interest can be visualized within days, without any prior understanding of the community. While the generation of GenomeFISH probes requires specialized equipment, existing isolate biobanks (i.e. ATCC and DSMZ) or stored SAGs provide accessible and immediate alternate resources for probe generation [53–60], with the Australian Human Microbiome Biobank (AHMB, https://ahmb.com.au/) planning to make GenomeFISH probes available for order in the near future. Given the superior sensitivity, phylogenetic resolution, and high-throughput nature of GenomeFISH, we envisage it will become widely used for the visualization of complex microbial communities.

## Supplementary Material

Figure_S1_wraf138

Figure_S2_wraf138

Figure_S3_wraf138

Figure_S4_wraf138

Figure_S5_wraf138

Figure_S6_wraf138

Figure_S7_wraf138

Figure_S8_wraf138

Figure_S9_wraf138

Figure_S10_wraf138

Figure_S11_wraf138

Figure_S12_wraf138

Figure_S13_wraf138

Figure_S14_wraf138

Table_S1_wraf138

Table_S2_wraf138

Table_S3_wraf138

Table_S4_wraf138

Supplementary_information_wraf138

## Data Availability

All data for this study, including metagenomic reads, isolate genomes, and MAGs can be found under Bioproject ID PRJNA1145114.

## References

[1] DeLong EF, Wickham GS, Pace NR. Phylogenetic stains: ribosomal RNA-based probes for the identification of single cells. *Science* 1989;243:1360–3. 10.1126/science.2466341 2466341

[2] Amann RI, Krumholz L, Stahl DA. Fluorescent-oligonucleotide probing of whole cells for determinative, phylogenetic, and environmental studies in microbiology. *J Bacteriol* 1990;172:762–70. 10.1128/jb.172.2.762-770.1990 1688842 PMC208504

[3] McIlroy SJ, Leu AO, Zhang X et al. Anaerobic methanotroph ‘*Candidatus* Methanoperedens nitroreducens’ has a pleomorphic life cycle. *Nat Microbiol* 2023;8:321–31. https://doi.org:10.10.1038/s41564-022-01292-9 36635574 10.1038/s41564-022-01292-9

[4] Daims H, Nielsen JL, Nielsen PH et al. In situ characterization of Nitrospira-like nitrite-oxidizing bacteria active in wastewater treatment plants. *Appl Environ Microbiol* 2001;67:5273–84. 10.1128/AEM.67.11.5273-5284.2001 11679356 PMC93301

[5] Langendijk PS, Schut F, Jansen GJ et al. Quantitative fluorescence *in situ* hybridization of *Bifidobacterium* spp. with genus-specific 16S rRNA-targeted probes and its application in fecal samples. *Appl Environ Microbiol* 1995;61:3069–75. 10.1128/aem.61.8.3069-3075.1995 7487040 PMC167584

[6] Ge X, Pereira FC, Mitteregger M et al. SRS-FISH: a high-throughput platform linking microbiome metabolism to identity at the single-cell level. *Proc Natl Acad Sci USA* 2022; 119:e2203519119. 10.1073/pnas.2203519119 35727976 PMC9245642

[7] Klotz F, Kitzinger K, Ngugi DK et al. Quantification of archaea-driven freshwater nitrification from single cell to ecosystem levels. *ISME J* 2022;16:1647–56. https://doi.org:10.10.1038/s41396-022-01216-9 35260828 10.1038/s41396-022-01216-9 PMC9122916

[8] Wagner M, Nielsen PH, Loy A et al. Linking microbial community structure with function: fluorescence *in situ* hybridization-microautoradiography and isotope arrays. *Curr Opin Biotech* 2006;17:83–91. 10.1016/j.copbio.2005.12.006 16377170

[9] Amann RI, Ludwig W, Schleifer K-H. Phylogenetic identification and *in situ* detection of individual microbial cells without cultivation. *Microb Rev* 1995;59:143–69. 10.1128/mr.59.1.143-169.1995 PMC239358 7535888

[10] Amann R, Fuchs BM. Single-cell identification in microbial communities by improved fluorescence *in situ* hybridization techniques. *Nat Rev Microbiol* 2008;6:339–48. https://doi.org:10.10.1038/nrmicro1888 18414500 10.1038/nrmicro1888

[11] Pernthaler A, Pernthaler J, Amann R. Fluorescence *in situ* hybridization and catalyzed reporter deposition for the identification of marine bacteria. *Appl Environ Microbiol* 2002;68:3094–101. 10.1128/AEM.68.6.3094-3101.2002 12039771 PMC123953

[12] Yamaguchi T, Kawakami S, Hatamoto M et al. *In situ* DNA-hybridization chain reaction (HCR): a facilitated *in situ* HCR system for the detection of environmental microorganisms. *Environ Microbiol* 2015;17:2532–41. 10.1111/1462-2920.12745 25523128

[13] Hoshino T, Yilmaz LS, Noguera DR et al. Quantification of target molecules needed to detect microorganisms by fluorescence *in situ* hybridization (FISH) and catalyzed reporter deposition-FISH. *Appl Environ Microbiol* 2008;74:5068–77. 10.1128/AEM.00208-08 18552182 PMC2519275

[14] Locey KJ, Lennon JT. Scaling laws predict global microbial diversity. *Proc Natl Acad Sci USA* 2016;113:5970–5. 10.1073/pnas.1521291113 27140646 PMC4889364

[15] Wang Q, Garrity George M, Tiedje James M et al. Naïve Bayesian classifier for rapid assignment of rRNA sequences into the new bacterial taxonomy. *Appl Environ Microbiol* 2007;73:5261–7. 10.1128/AEM.00062-07 17586664 PMC1950982

[16] Lanoil BD, Giovannoni SJ. Identification of bacterial cells by chromosomal painting. *Appl Environ Microbiol* 1997;63:1118–23. 10.1128/aem.63.3.1118-1123.1997 9055426 PMC168401

[17] Fuchs BM, Pernthaler J, Amann R. Methods for General and Molecular Microbiology 886–896. Washington, DC: ASM Press, 2007.

[18] Haroon MF, Hu S, Shi Y et al. Anaerobic oxidation of methane coupled to nitrate reduction in a novel archaeal lineage. *Nature* 2013;500:567–70. 10.1038/nature12375 23892779

[19] Teh J, Berendsen EM, Hoedt EC et al. Novel strain-level resolution of Crohn’s disease mucosa-associated microbiota via an ex vivo combination of microbe culture and metagenomic sequencing. *ISME J* 2021;15:3326–38. 10.1038/s41396-021-00991-1 34035441 PMC8528831

[20] Rinke, C. Single-cell genomics of microbial dark matter. Methods Mol Biol, 1849:99–111, (2018), New York, NY, Springer New York, 10.1007/978-1-4939-8728-3_7 30298250

[21] Newell RJP, Aroney STN, Zaugg J et al. Aviary: hybrid assembly and genome recovery from metagenomes with aviary (v0.9.0). *Zenodo* 2024. https://doi.org:10.https://doi.org/10.5281/zenodo.10806928

[22] Nurk S, Meleshko D, Korobeynikov A et al. metaSPAdes: a new versatile metagenomic assembler. *Genome Res* 2017;27:824–34. 10.1101/gr.213959.116 28298430 PMC5411777

[23] Kang DD, Froula J, Egan R et al. MetaBAT, an efficient tool for accurately reconstructing single genomes from complex microbial communities. *PeerJ* 2015;3:e1165. 10.7717/peerj.1165 26336640 PMC4556158

[24] Kang DD, Li F, Kirton E et al. MetaBAT 2: an adaptive binning algorithm for robust and efficient genome reconstruction from metagenome assemblies. *PeerJ* 2019;7:e7359. 10.7717/peerj.7359 31388474 PMC6662567

[25] Wu Y-W, Simmons BA, Singer SW. MaxBin 2.0: an automated binning algorithm to recover genomes from multiple metagenomic datasets. *Bioinformatics* 2015;32:605–7. 10.1093/bioinformatics/btv638 26515820

[26] Alneberg J, Bjarnason BS, de Bruijn I et al. Binning metagenomic contigs by coverage and composition. *Nat Methods* 2014;11:1144–6. https://doi.org:10.10.1038/nmeth.3103 25218180 10.1038/nmeth.3103

[27] Nissen JN, Johansen J, Allesøe RL et al. Improved metagenome binning and assembly using deep variational autoencoders. *Nat Biotechnol* 2021;39:555–60. https://doi.org:10.10.1038/s41587-020-00777-4 33398153 10.1038/s41587-020-00777-4

[28] Pan S, Zhao X-M, Coelho LP. SemiBin2: self-supervised contrastive learning leads to better MAGs for short- and long-read sequencing. *Bioinform* 2023;39:i21–9. 10.1093/bioinformatics/btad209 PMC10311329 37387171

[29] Newell RJP, Tyson GW, Woodcroft BJ. Rosella: metagenomic binning using UMAP and HDBSCAN (v0.5.4). *Zenodo* 2024. https://doi.org:10.https://doi.org/10.5281/zenodo.12753160

[30] Sieber CMK, Probst AJ, Sharrar A et al. Recovery of genomes from metagenomes via a dereplication, aggregation and scoring strategy. *Nat Microbiol* 2018;3:836–43. https://doi.org:10.10.1038/s41564-018-0171-1 29807988 10.1038/s41564-018-0171-1 PMC6786971

[31] Chklovski A, Parks DH, Woodcroft BJ et al. CheckM2: a rapid, scalable and accurate tool for assessing microbial genome quality using machine learning. *Nat Methods* 2023;20:1203–12. https://doi.org:10.10.1038/s41592-023-01940-w 37500759 10.1038/s41592-023-01940-w

[32] Bowers RM et al. Minimum information about a single amplified genome (MISAG) and a metagenome-assembled genome (MIMAG) of bacteria and archaea. *Nat Biotechnol* 2017;35:725–31. 10.1038/nbt.3893 28787424 PMC6436528

[33] Chaumeil P-A, Mussig AJ, Hugenholtz P et al. GTDB-Tk v2: memory friendly classification with the genome taxonomy database. *Bioinform* 2022;38:5315–6. 10.1093/bioinformatics/btac672 PMC9710552 36218463

[34] Aroney ST, Newell RJ, Nissen JN et al. CoverM: read alignment statistics for metagenomics. *Bioinform* 2025;41:btaf147. 10.1093/bioinformatics/btaf147 PMC11993303 40193404

[35] Jain C, Rodriguez-R LM, Phillippy AM et al. High throughput ANI analysis of 90K prokaryotic genomes reveals clear species boundaries. *Nat Commun* 2018;9:5114. 10.1038/s41467-018-07641-9 30504855 PMC6269478

[36] Shaw J, Yu YW. Fast and robust metagenomic sequence comparison through sparse chaining with skani. *Nat Methods* 2023;20:1661–5. 10.1038/s41592-023-02018-3 37735570 PMC10630134

[37] Barrero-Canosa J, Moraru C, Zeugner L et al. Direct-geneFISH: a simplified protocol for the simultaneous detection and quantification of genes and rRNA in microorganisms. *Environ Microbiol* 2017;19:70–82. 10.1111/1462-2920.13432 27348074

[38] Manz W, Amann R, Ludwig W et al. Phylogenetic oligodeoxynucleotide probes for the major subclasses of proteobacteria: problems and solutions. *Syst Appl Microbiol* 1992;15:593–600. 10.1016/S0723-2020(11)80121-9

[39] Amann RI, Binder BJ, Olson RJ et al. Combination of 16S rRNA-targeted oligonucleotide probes with flow cytometry for analyzing mixed microbial populations. *Appl Environ Microbiol* 1990;56:1919–25. 10.1128/aem.56.6.1919-1925.1990 2200342 PMC184531

[40] Schmid M, Schmitz-Esser S, Jetten M et al. 16S-23S rDNA intergenic spacer and 23S rDNA of anaerobic ammonium-oxidizing bacteria: implications for phylogeny and *in situ* detection. *Environ Microbiol* 2001;3:450–9. 10.1046/j.1462-2920.2001.00211.x 11553235

[41] Stirling DR, Swain-Bowden MJ, Lucas AM et al. CellProfiler 4: improvements in speed, utility and usability. *BMC Bioinform* 2021;22:433. 10.1186/s12859-021-04344-9 PMC8431850 34507520

[42] Li CH, Lee C. Minimum cross entropy thresholding. *Pattern Recogn* 1993;26:617–25. 10.1016/0031-3203(93)90115-D

[43] Schindelin J, Arganda-Carreras I, Frise E et al. Fiji: an open-source platform for biological-image analysis. *Nat Methods* 2012;9:676–82. 10.1038/nmeth.2019 22743772 PMC3855844

[44] R Core Team . R: a language and environment for statistical computing. 2013.

[45] Ogle DH J.D, Wheeler AP et al. FSA: simple fisheries stock assessment methods. *R package version* 2025;0.9.6: https://doi.org:10.https://fishr-core-team.github.io/FSA/

[46] Bremer H, Dennis PP. Modulation of chemical composition and other parameters of the cell by growth rate. *EcoSal Plus* 1996;2:1553–69. 10.1128/ecosal.5.2.3 26443740

[47] Lightfield J, Fram NR, Ely B. Across bacterial phyla, distantly-related genomes with similar genomic GC content have similar patterns of amino acid usage. *PLoS One* 2011;6:e17677. 10.1371/journal.pone.0017677 21423704 PMC3053387

[48] Luef B, Frischkorn KR, Wrighton KC et al. Diverse uncultivated ultra-small bacterial cells in groundwater. *Nat Commun* 2015;6:6372. https://doi.org:10.10.1038/ncomms7372 25721682 10.1038/ncomms7372

[49] Chiriac M-C, Bulzu PA, Andrei AS et al. Ecogenomics sheds light on diverse lifestyle strategies in freshwater CPR. *Microbiome* 2022;10:84. https://doi.org:10.10.1186/s40168-022-01274-3 35659305 10.1186/s40168-022-01274-3 PMC9166423

[50] Blagodatskaya E, Kuzyakov Y. Active microorganisms in soil: critical review of estimation criteria and approaches. *Soil Biol Biochem* 2013;67:192–211. 10.1016/j.soilbio.2013.08.024

[51] Wada N, Pollock FJ, Willis BL et al. *In situ* visualization of bacterial populations in coral tissues: pitfalls and solutions. *PeerJ* 2016;4:e2424. 10.7717/peerj.2424 27688961 PMC5036075

[52] Yuan C, Lei J, Cole J et al. Reconstructing 16S rRNA genes in metagenomic data. *Bioinform* 2015;31:i35–43. 10.1093/bioinformatics/btv231 PMC4765874 26072503

[53] Huang Y, Sheth RU, Zhao S et al. High-throughput microbial culturomics using automation and machine learning. *Nat Biotechnol* 2023;41:1424–33. https://doi.org:10.10.1038/s41587-023-01674-2 36805559 10.1038/s41587-023-01674-2 PMC10567565

[54] Seshadri R et al. Cultivation and sequencing of rumen microbiome members from the Hungate1000 collection. *Nat Biotechnol* 2018;36:359–67. 10.1038/nbt.4110 29553575 PMC6118326

[55] Afrizal A, Jennings SAV, Hitch TCA et al. Enhanced cultured diversity of the mouse gut microbiota enables custom-made synthetic communities. *Cell Host Microbe* 2022;30:1630–1645.e25. https://doi.org:10.10.1016/j.chom.2022.09.011 36208631 10.1016/j.chom.2022.09.011

[56] Ide K, Nishikawa Y, Maruyama T et al. Targeted single-cell genomics reveals novel host adaptation strategies of the symbiotic bacteria Endozoicomonas in *Acropora tenuis* coral. *Microbiome* 2022;10:220. https://doi.org:10.10.1186/s40168-022-01395-9 36503599 10.1186/s40168-022-01395-9 PMC9743535

[57] Hosokawa M, Nishikawa Y, Kogawa M et al. Massively parallel whole genome amplification for single-cell sequencing using droplet microfluidics. *Sci Rep* 2017;7:5199. 10.1038/s41598-017-05436-4 28701744 PMC5507899

[58] Aoki W, Kogawa M, Matsuda S et al. Massively parallel single-cell genomics of microbiomes in rice paddies. *Front Microbiol* 2022;13:1024640. 10.3389/fmicb.2022.1024640 36406415 PMC9669790

[59] Anstett J, Plominsky AM, DeLong EF et al. A compendium of bacterial and archaeal single-cell amplified genomes from oxygen deficient marine waters. *Sci Data* 2023;10:332. 10.1038/s41597-023-02222-y 37244914 PMC10224968

[60] Rinke C, Schwientek P, Sczyrba A et al. Insights into the phylogeny and coding potential of microbial dark matter. *Nature* 2013;499:431–7. https://doi.org:10.10.1038/nature12352 23851394 10.1038/nature12352

