# Supplementary figures and images for: GenomeFISH: genome-based fluorescence in situ hybridization for strain-level visualization of microbial communities
Source: ISME J. 2025 Jul 7;19(1):wraf138. doi: 10.1093/ismejo/wraf138 (PMC12344553; doi:10.1093/ismejo/wraf138)

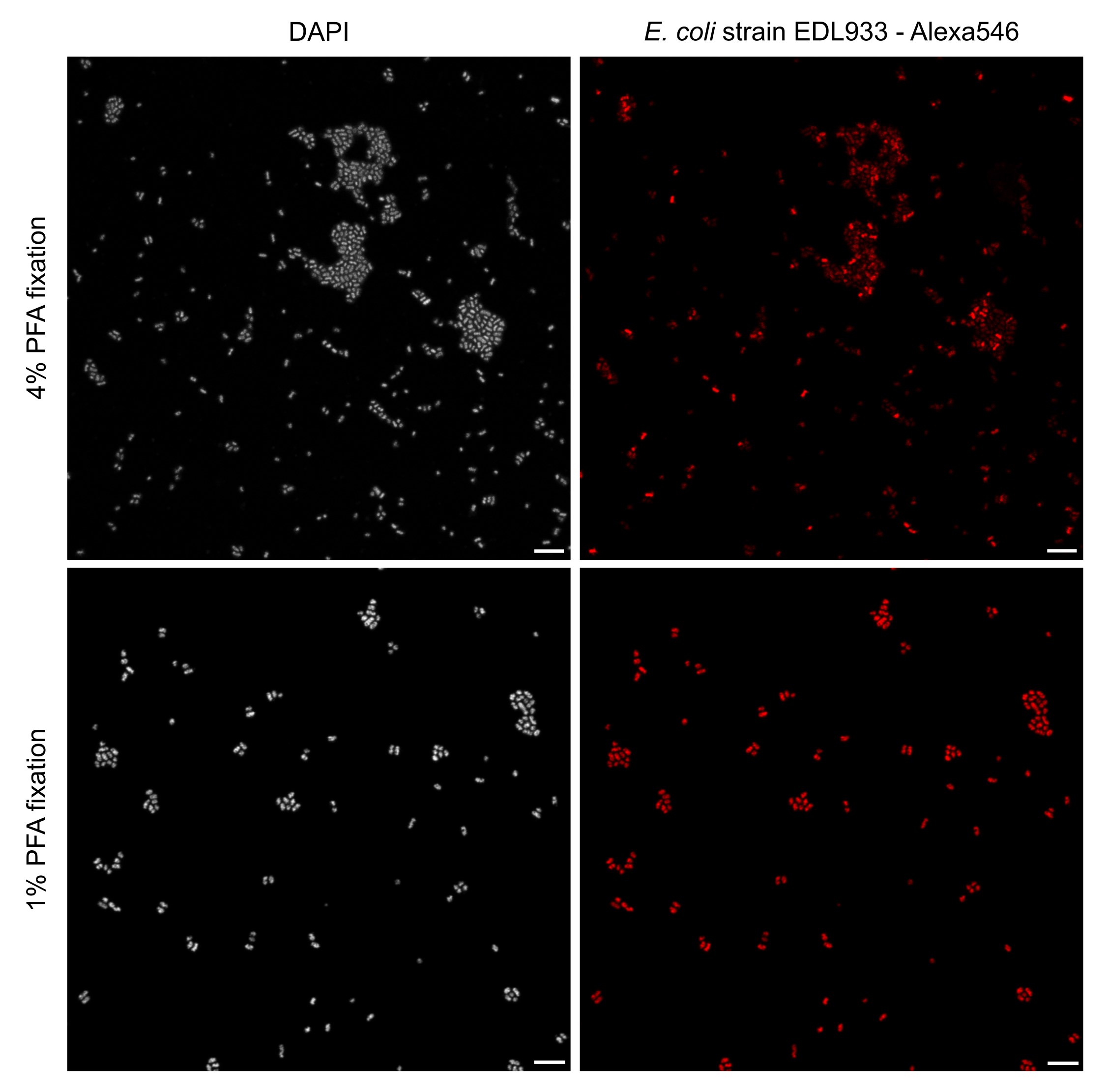

Supplement: Figure_S1_wraf138 [file figure_s1_wraf138.jpeg]

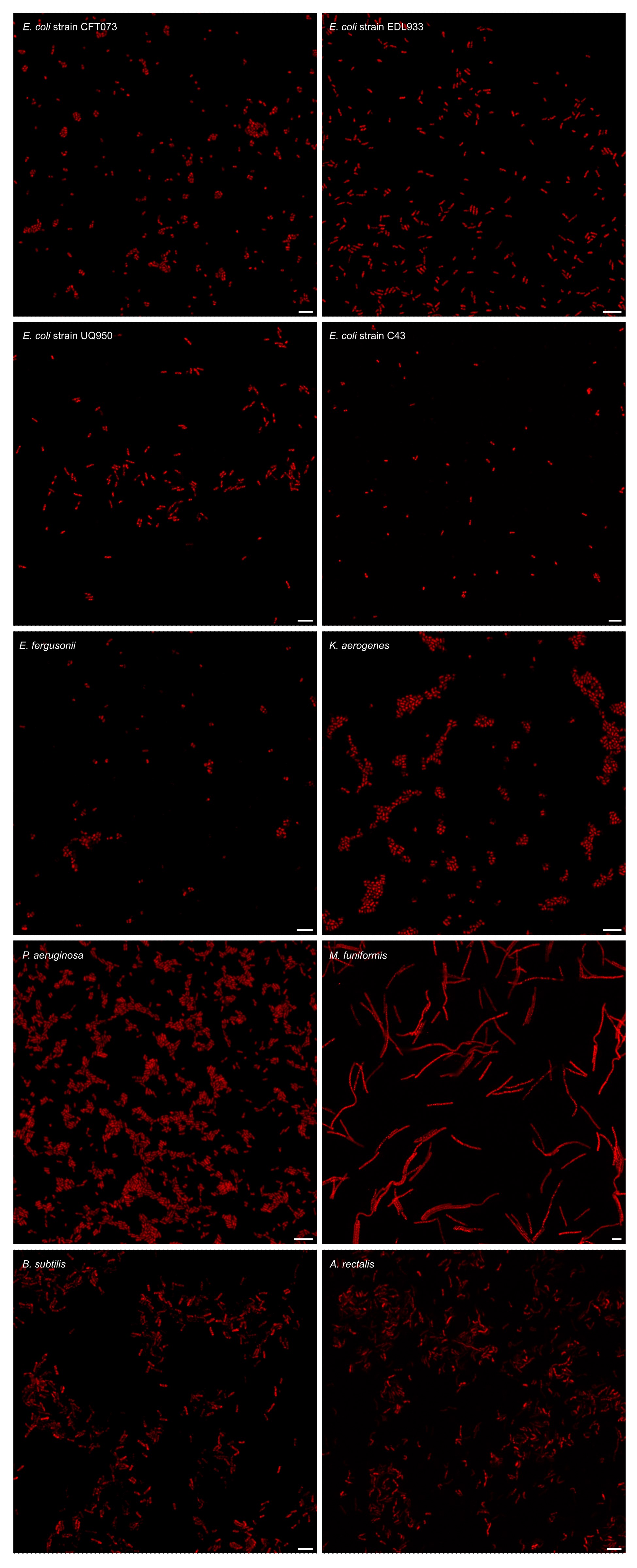

Supplement: Figure_S2_wraf138 [file figure_s2_wraf138.jpeg]

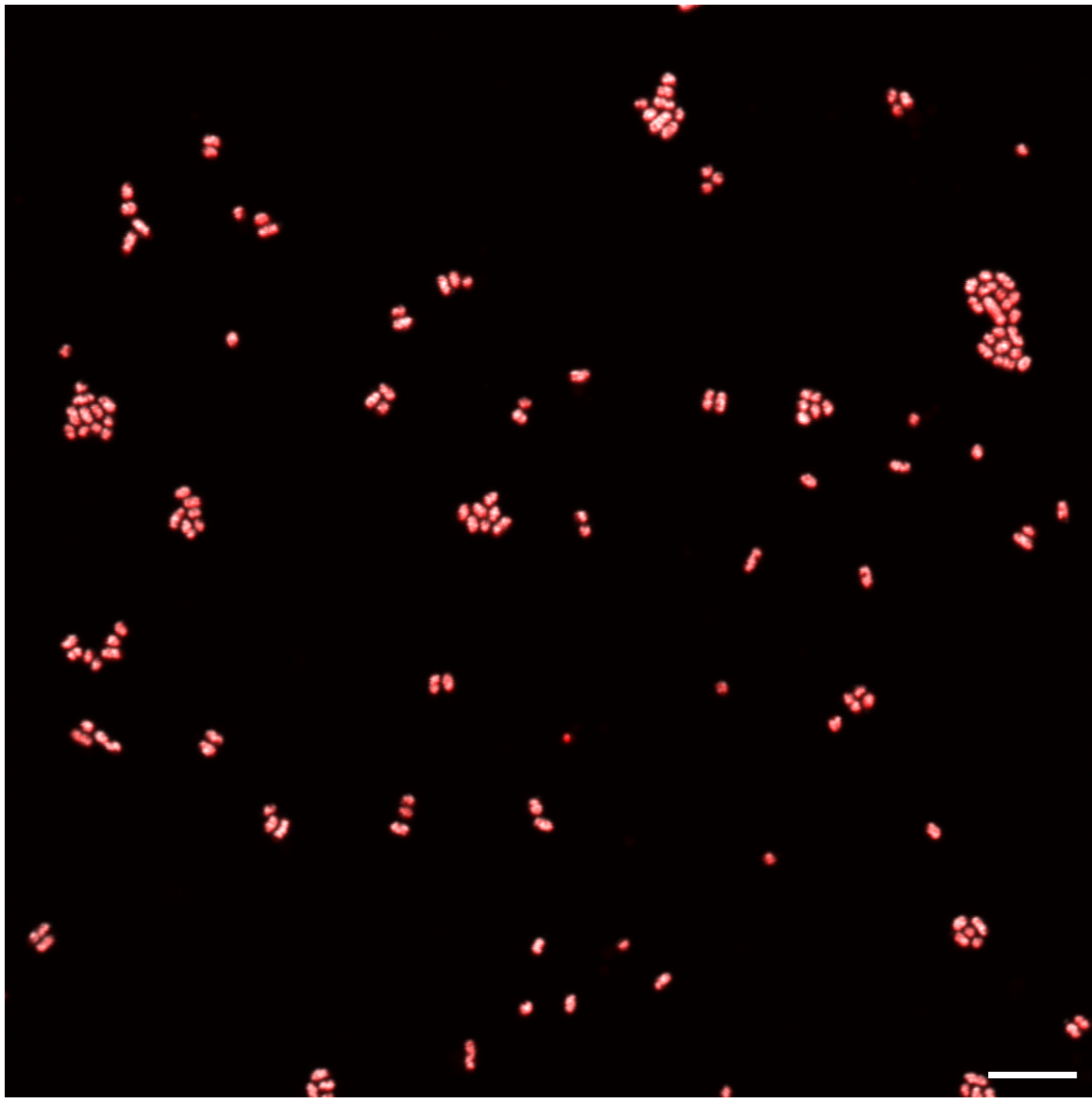

*E. coli* strain EDL933  
*E. coli* EDL933 probe

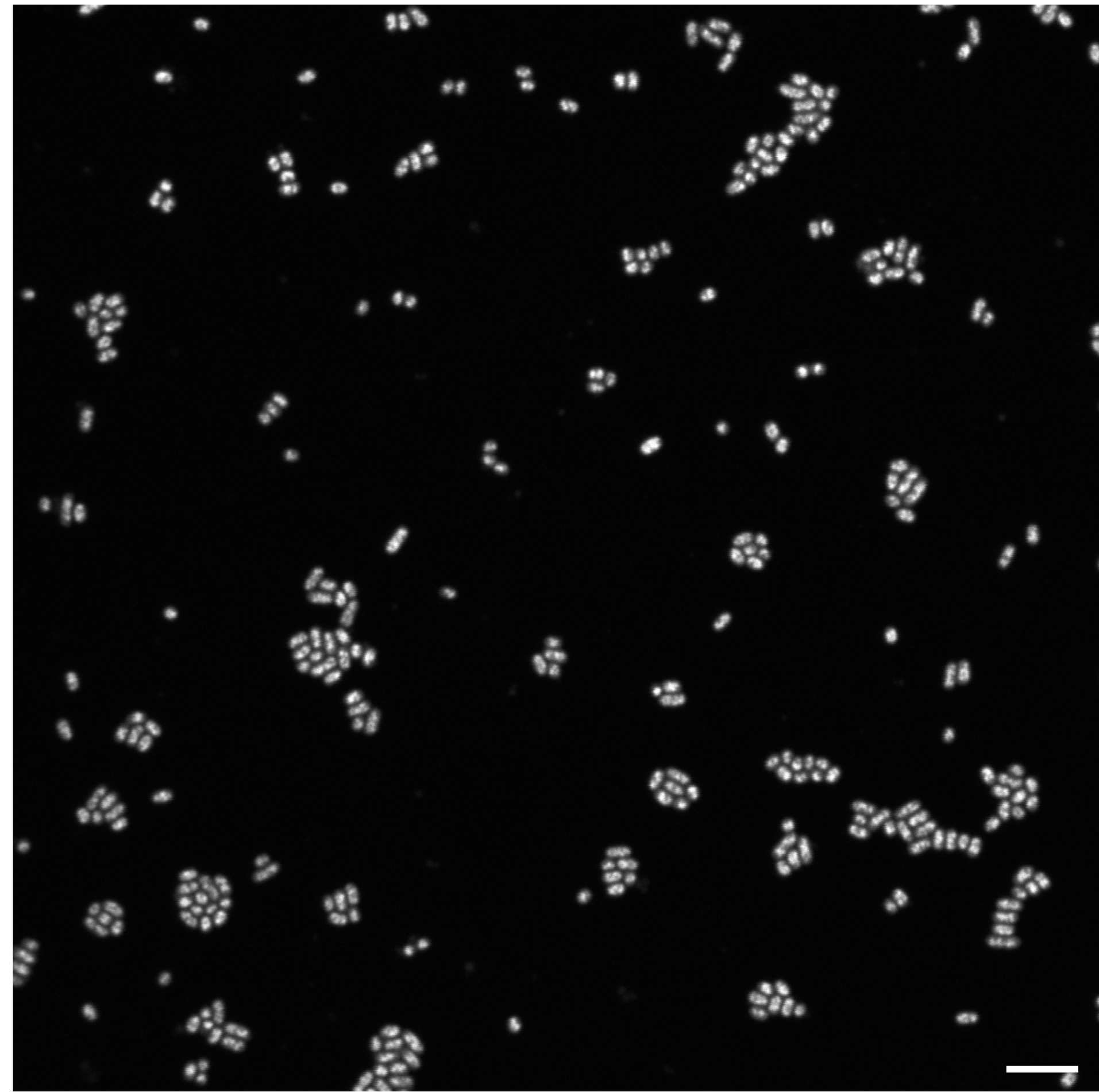

*E. coli* strain EDL933  
calf thymus DNA probe

Supplement: Figure_S3_wraf138 [file figure_s3_wraf138.pdf]

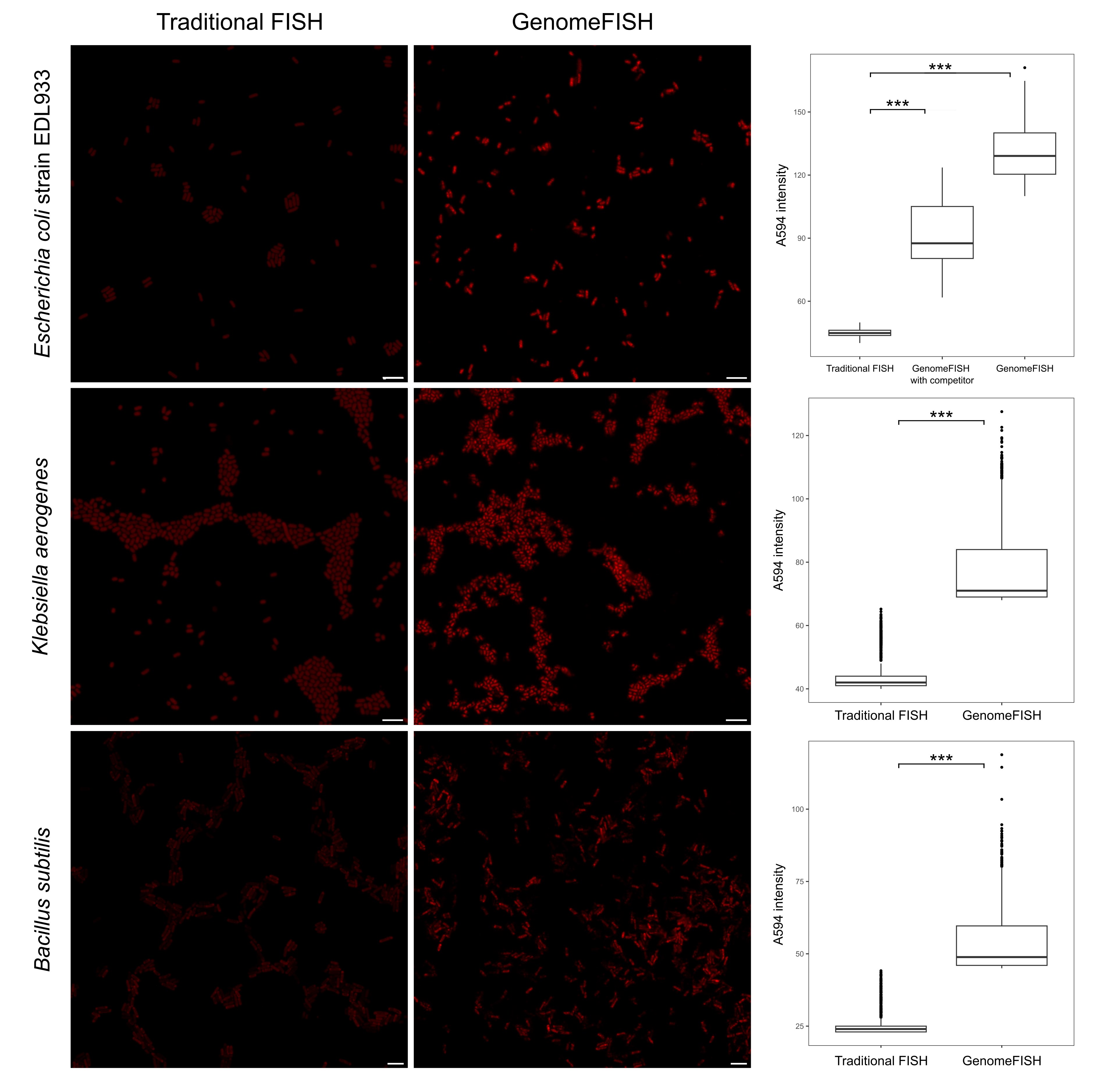

Supplement: Figure_S4_wraf138 [file figure_s4_wraf138.jpeg]

**A**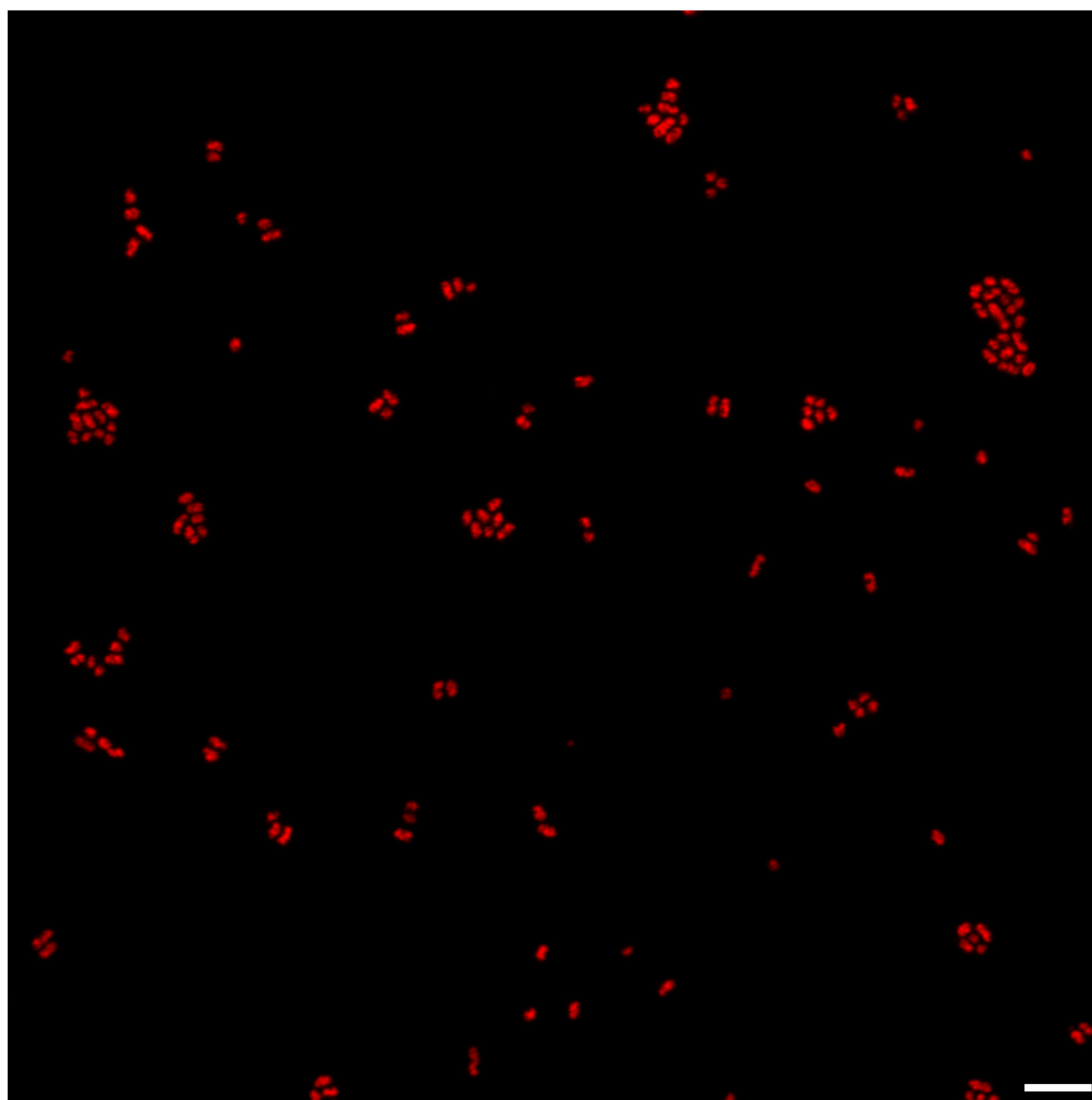

*E. coli* strain EDL933 - Alexa546

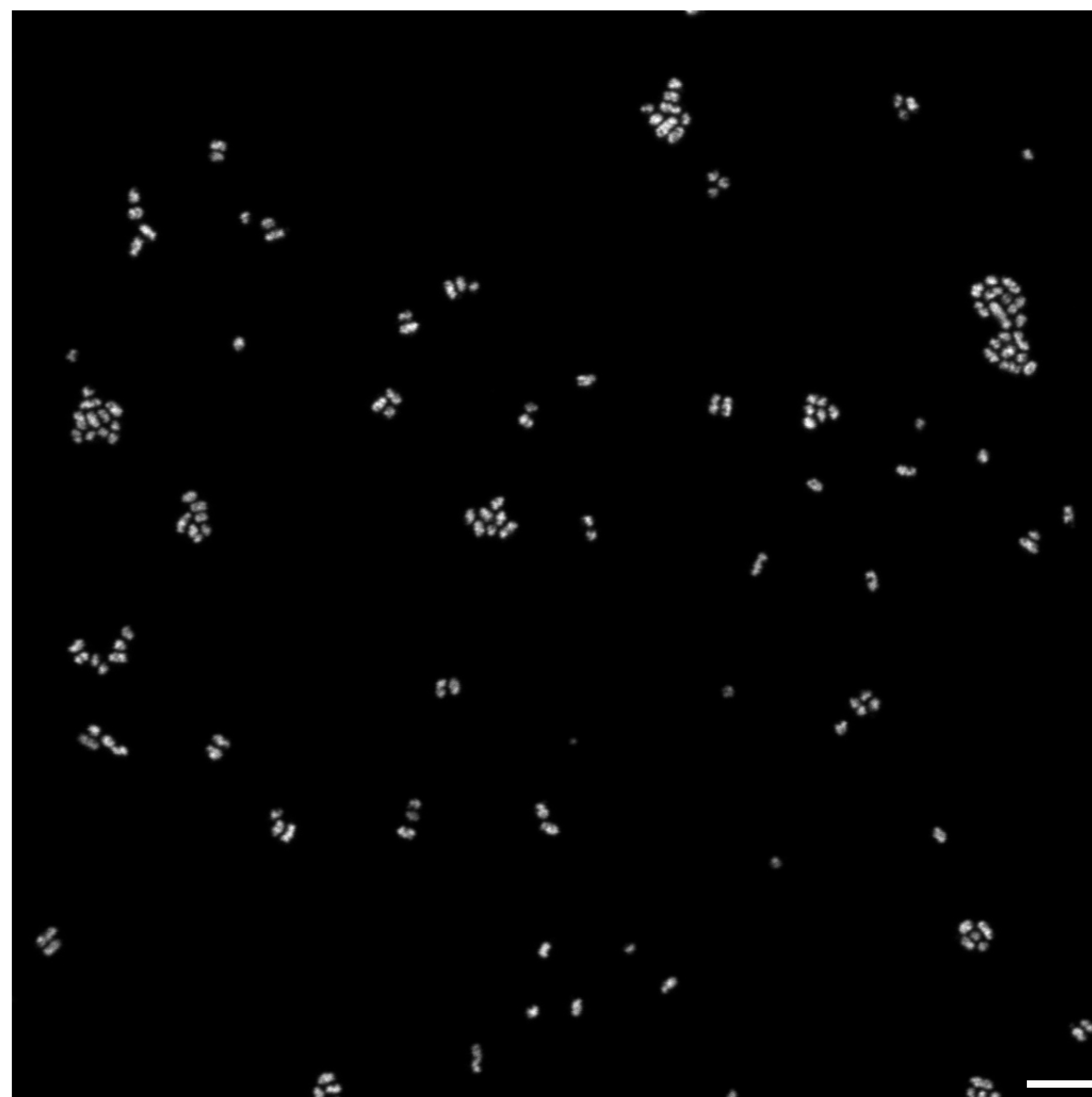

DAPI

**B**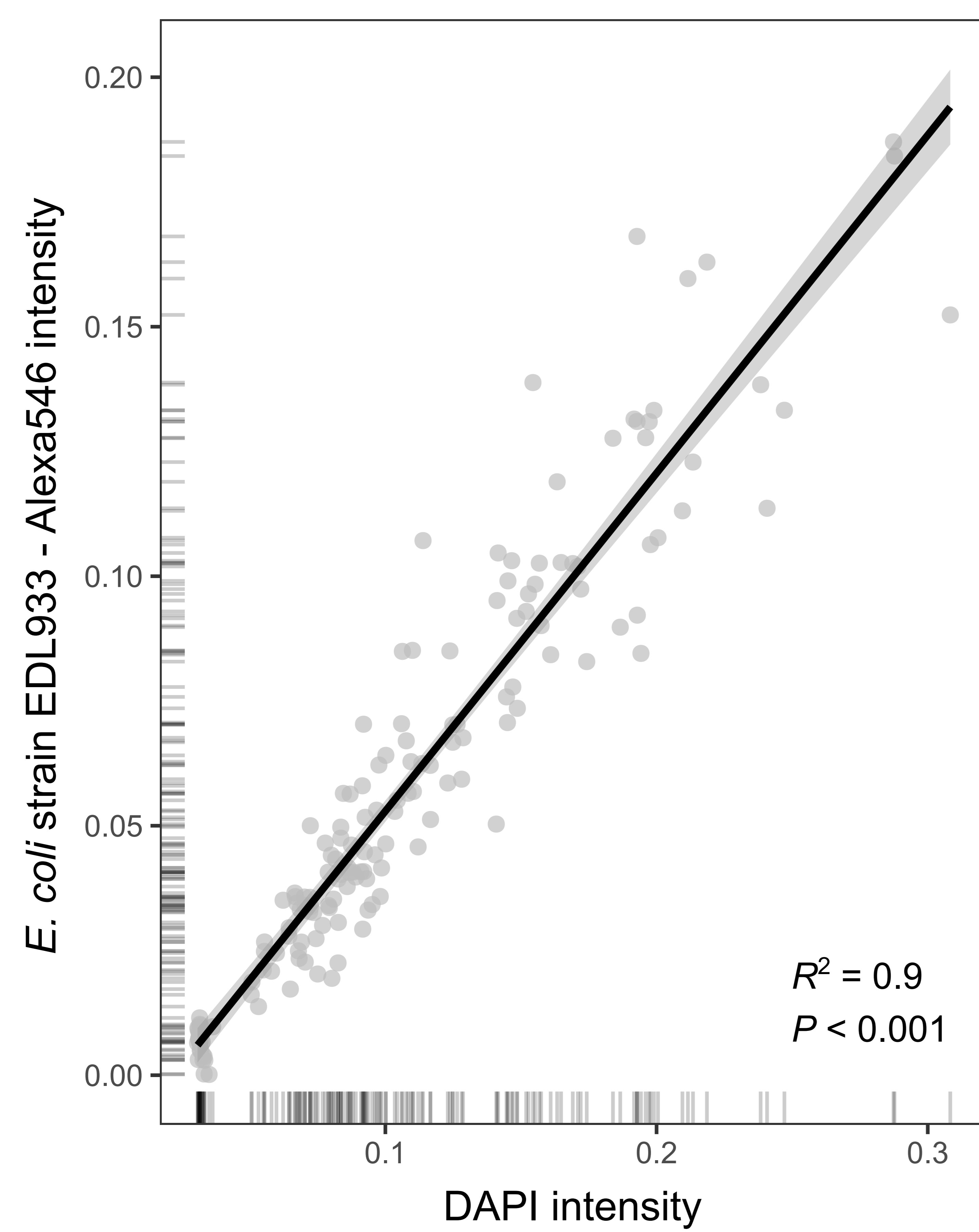**C**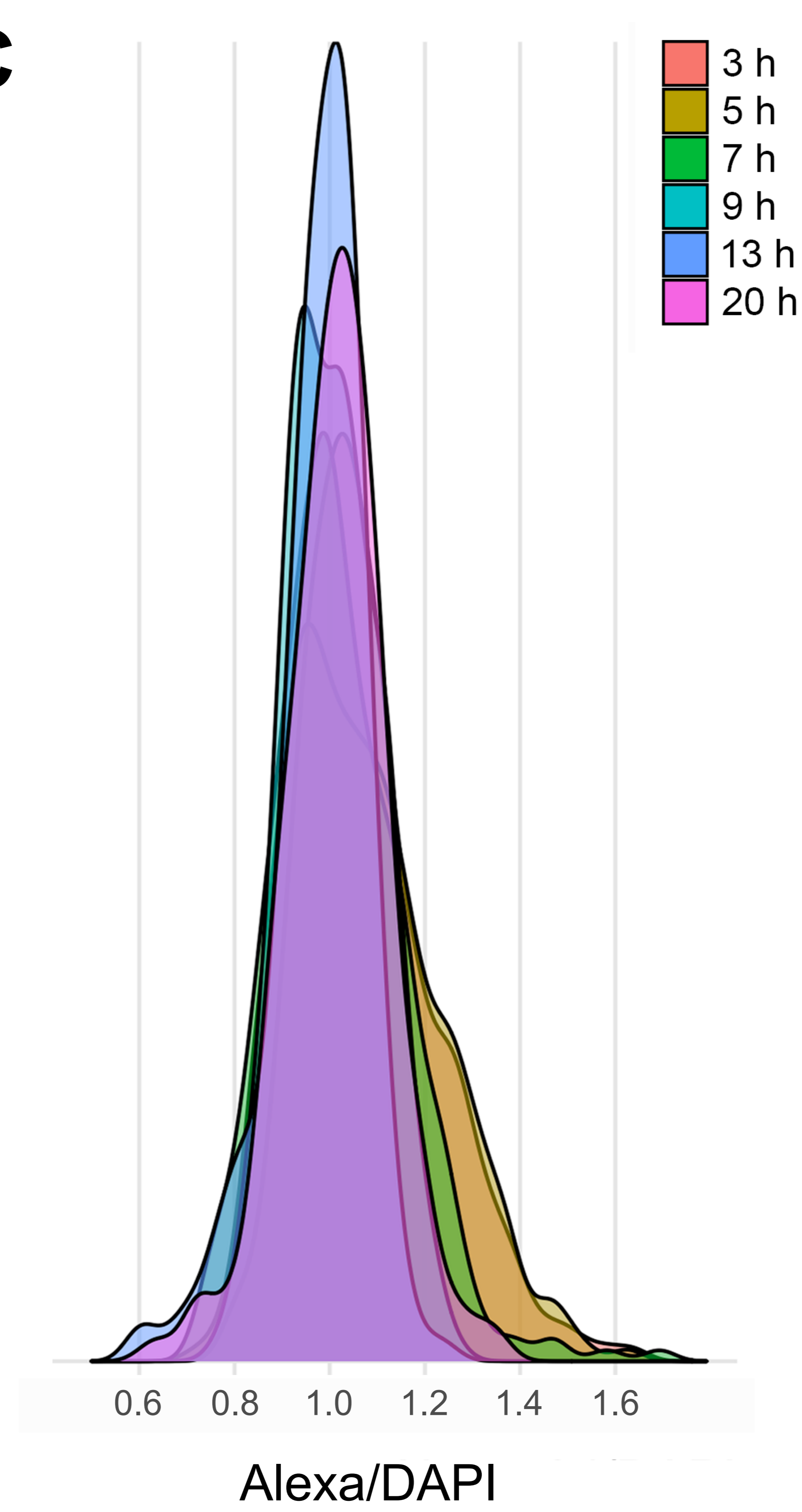**D**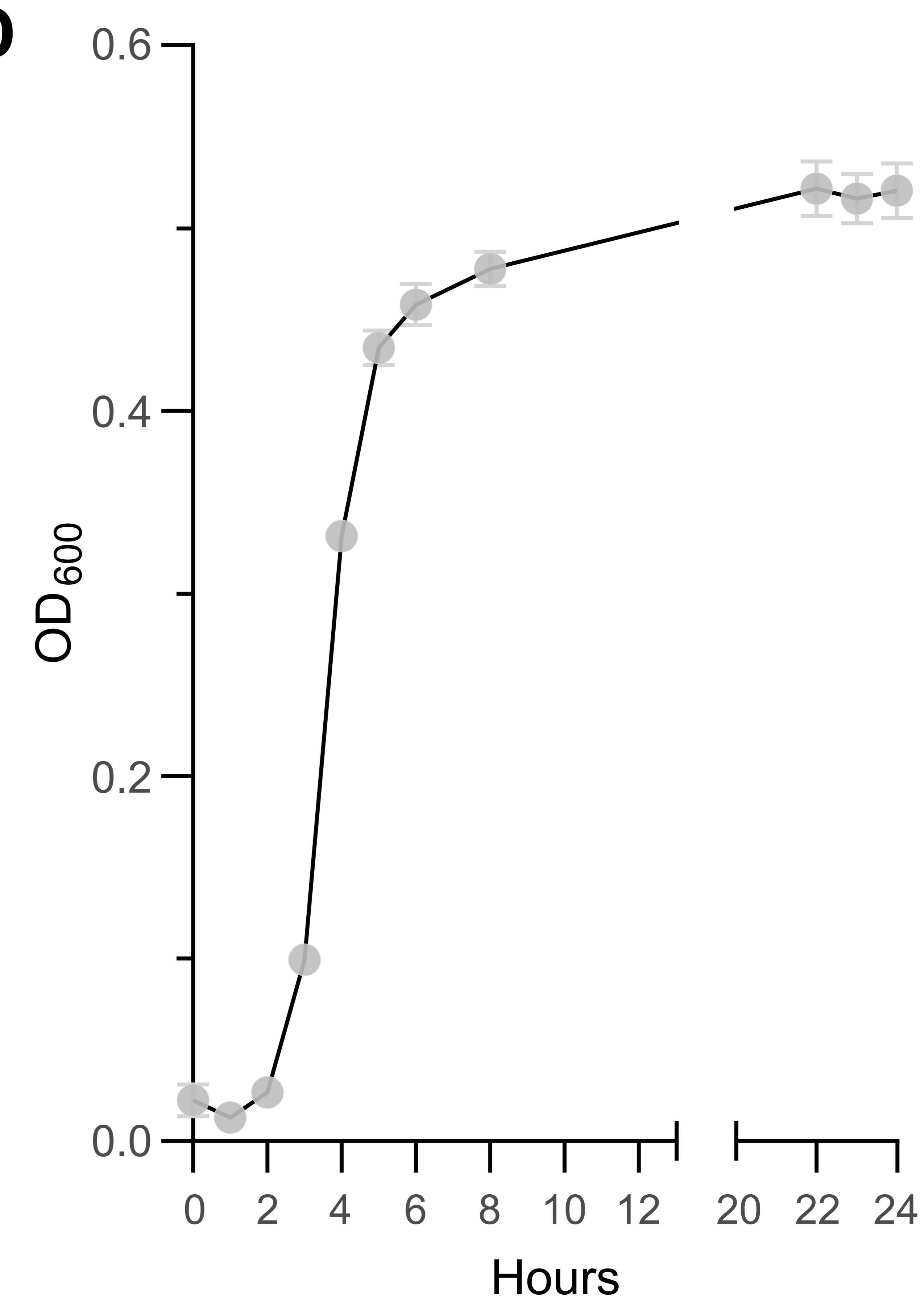

Supplement: Figure_S6_wraf138 [file figure_s6_wraf138.pdf]

Alexa594 RFU

*E. coli* strain EDL933

*E. fergusonii*

*E. coli* strain CFT073

*P. aeruginosa*

Alexa546 RFU

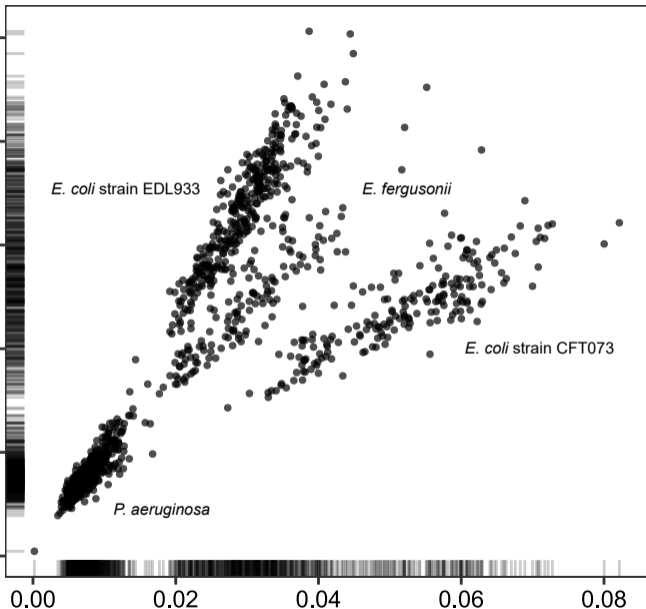

Supplement: Figure_S7_wraf138 [file figure_s7_wraf138.pdf]

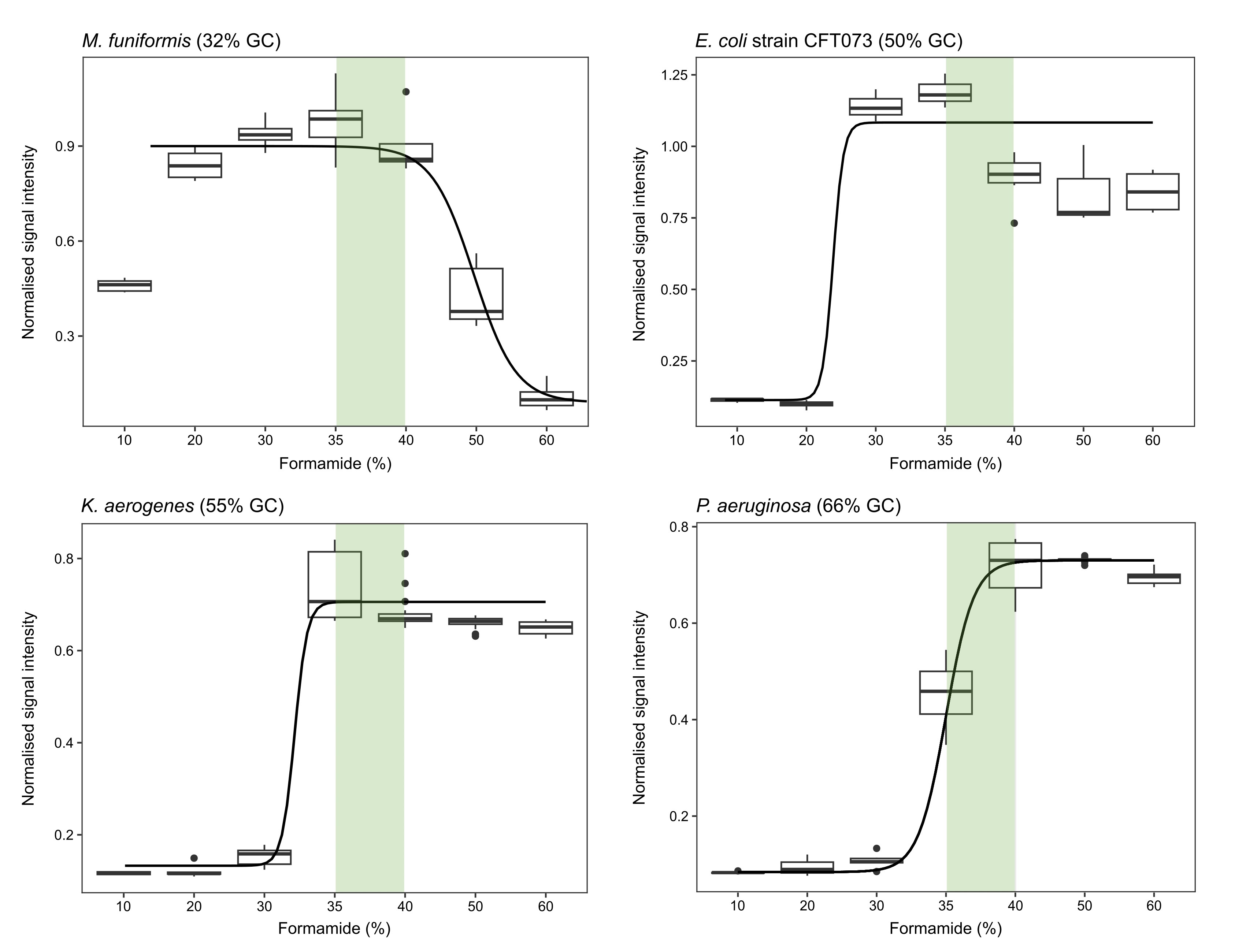

Supplement: Figure_S8_wraf138 [file figure_s8_wraf138.jpeg]

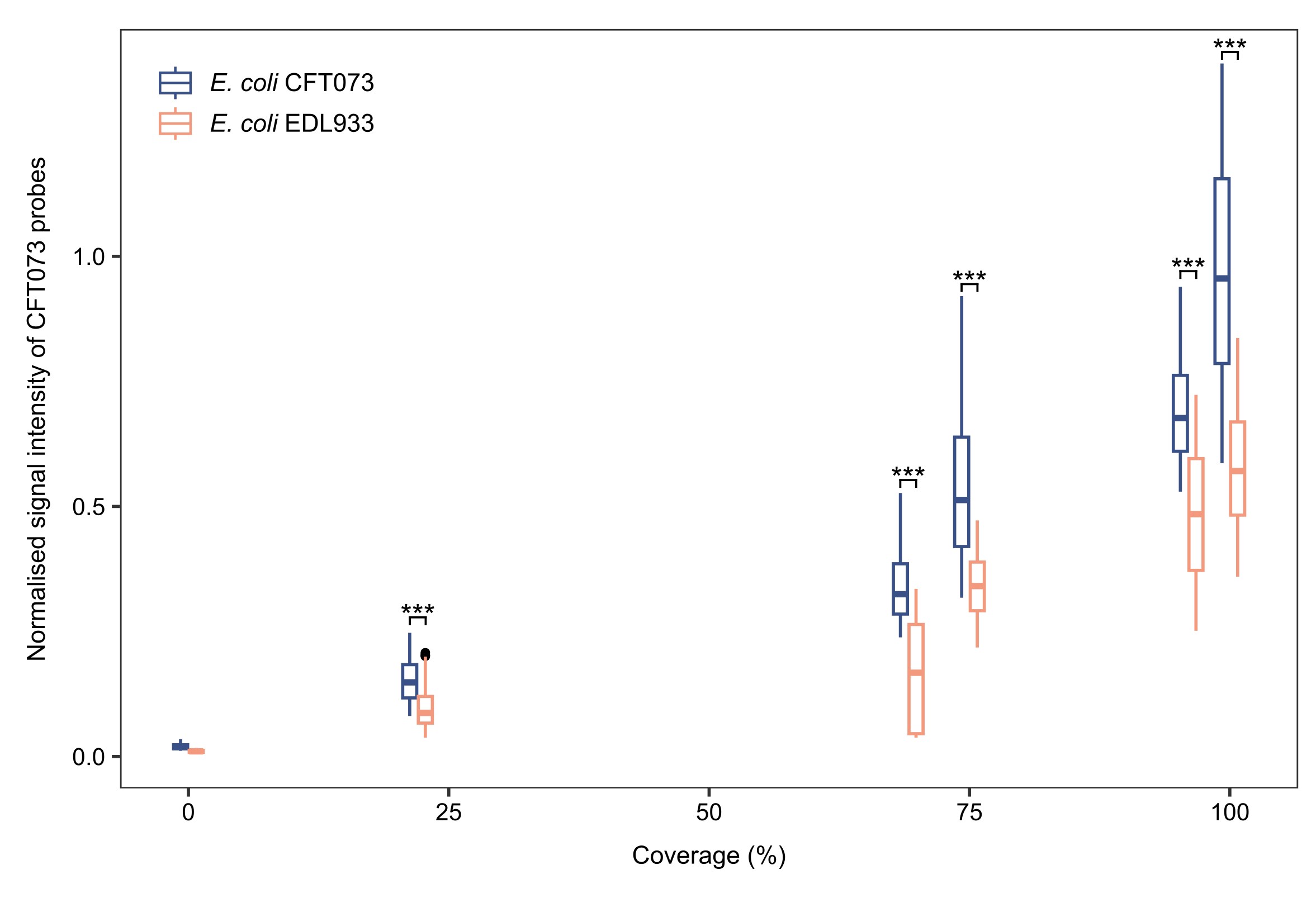

Supplement: Figure_S9_wraf138 [file figure_s9_wraf138.jpeg]

"Ca. M. nitroreducens"

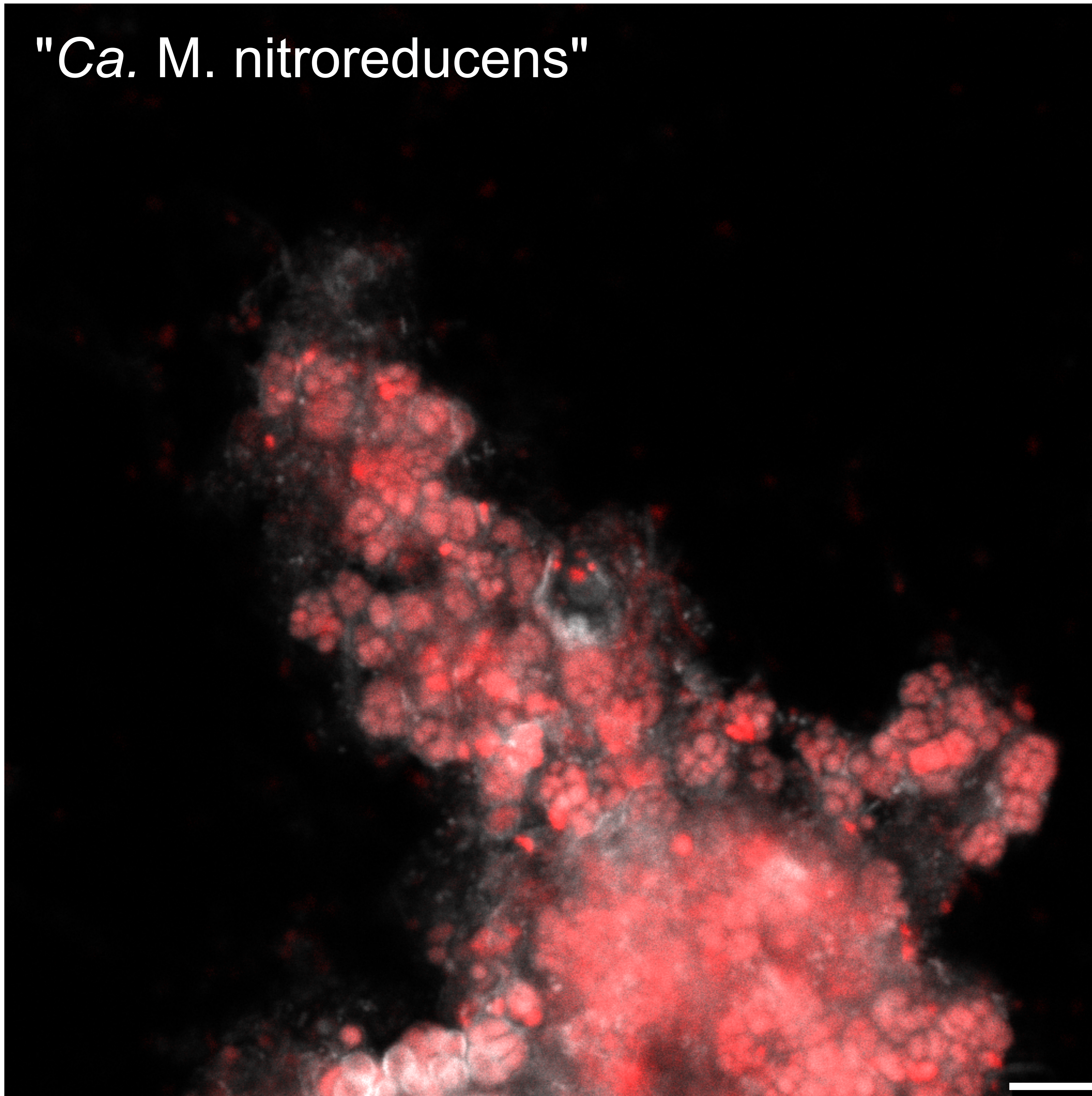

"Ca. K. stuttgartiensis"

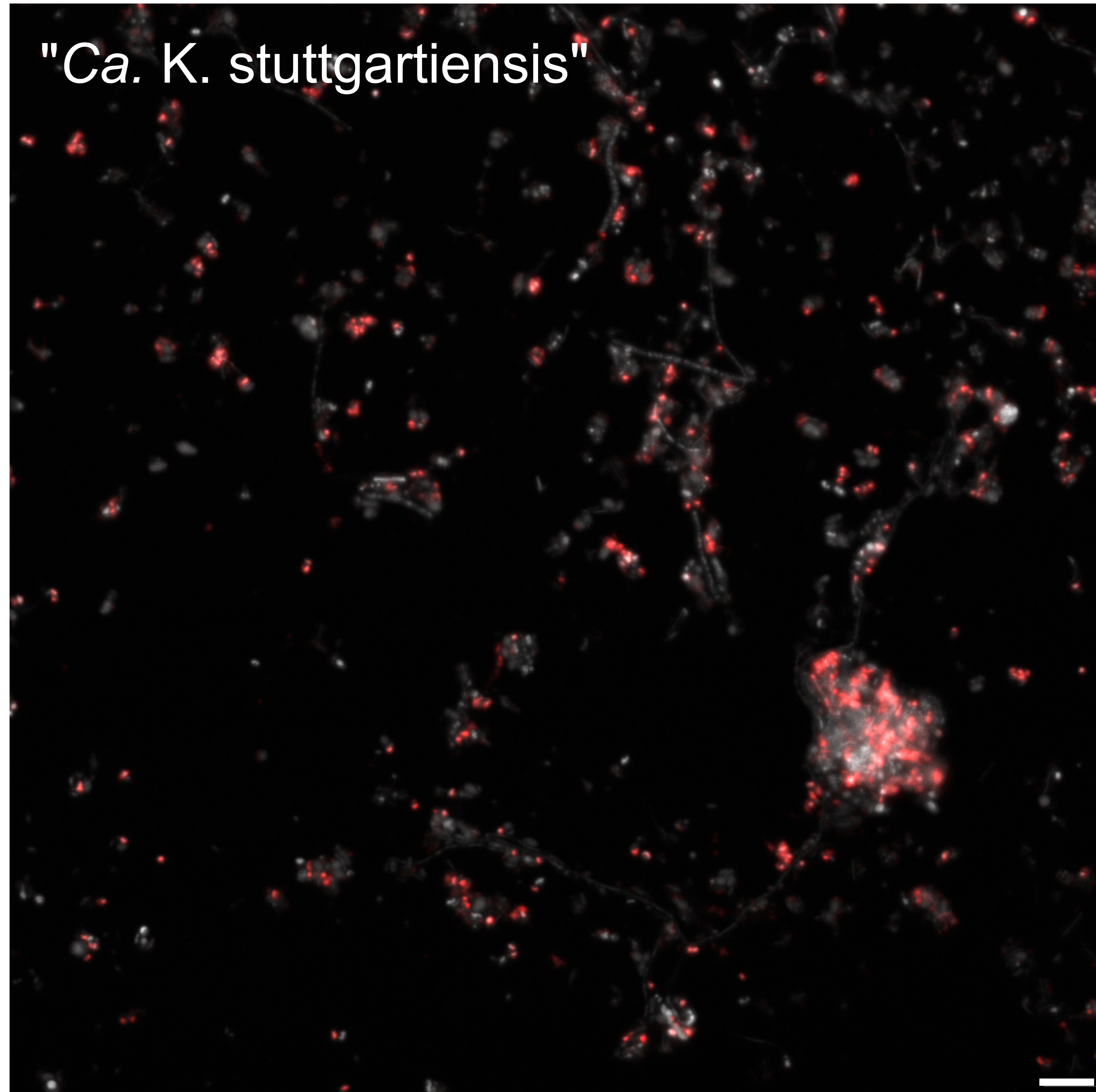

*S. sp003599395*

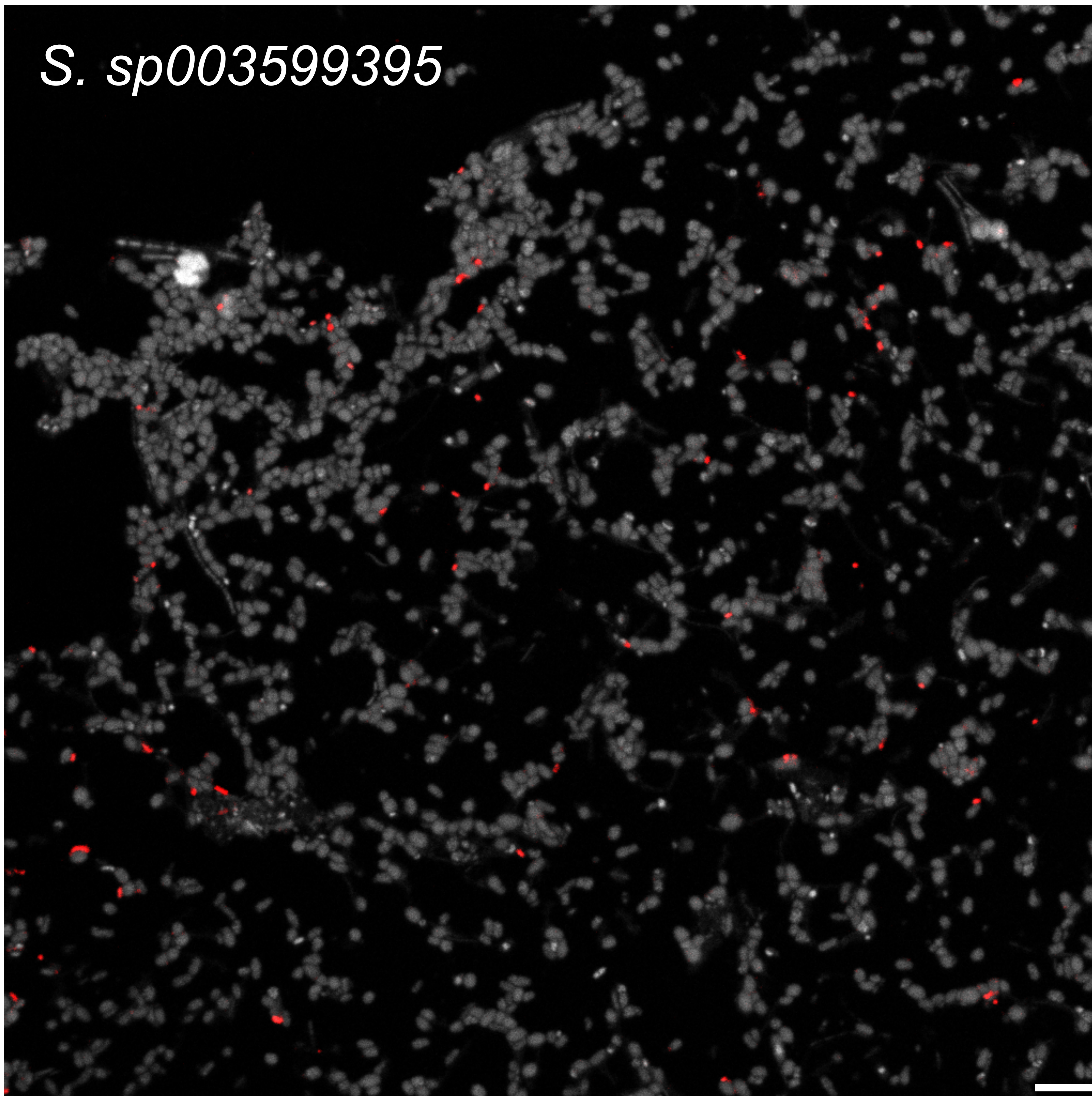

"Ca. C. aquaticus"

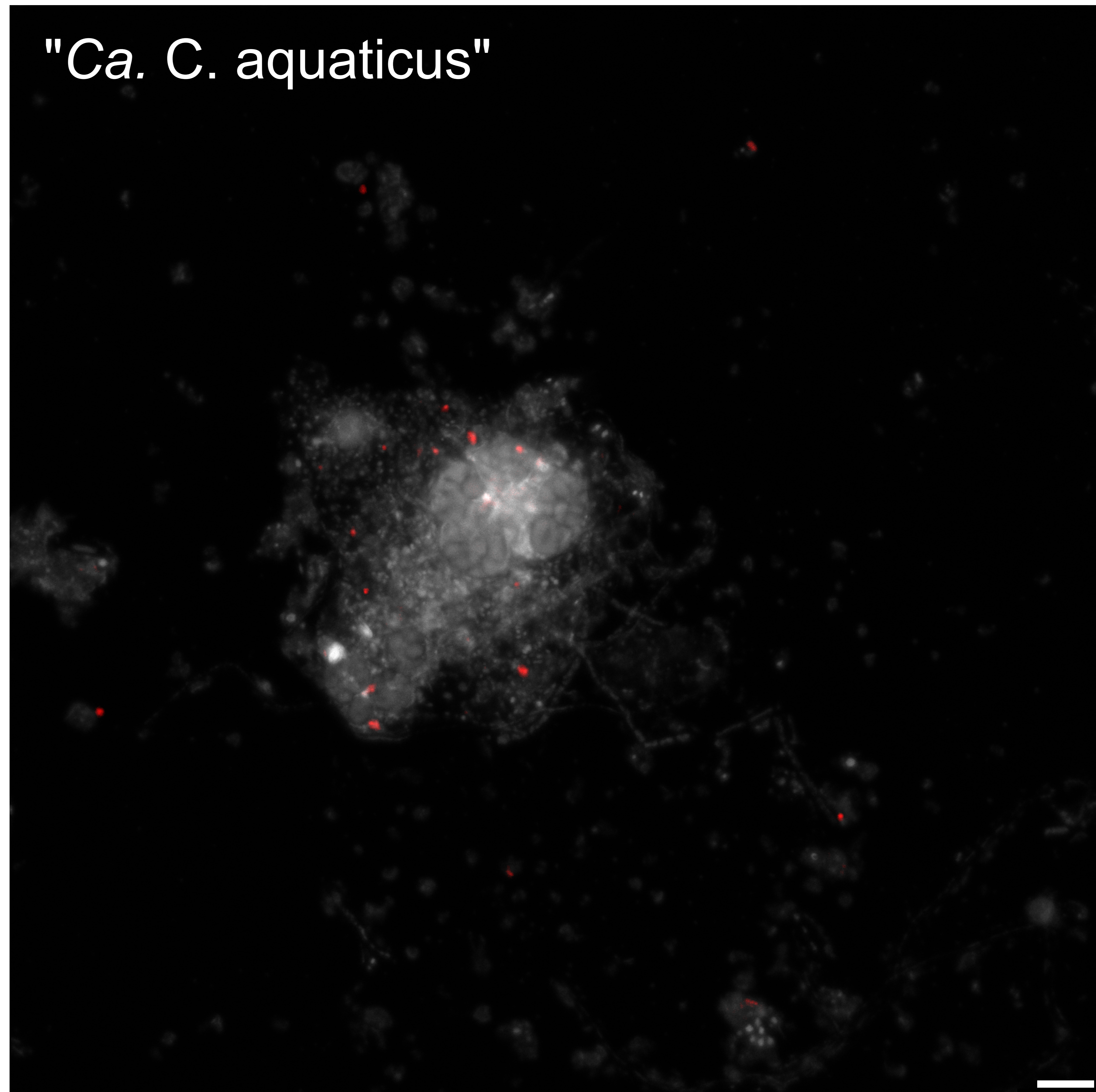

Supplement: Figure_S10_wraf138 [file figure_s10_wraf138.pdf]

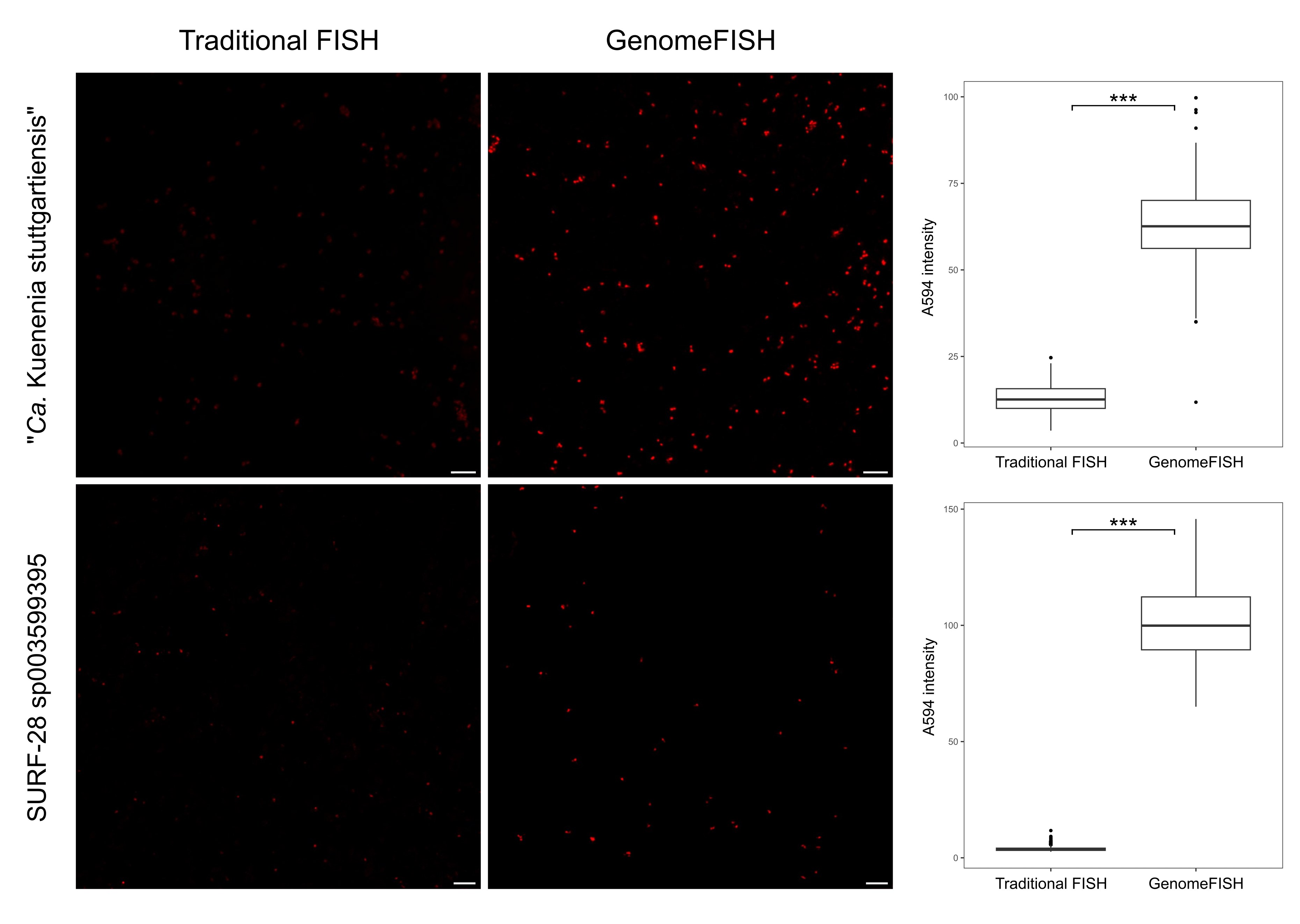

Supplement: Figure_S11_wraf138 [file figure_s11_wraf138.jpeg]

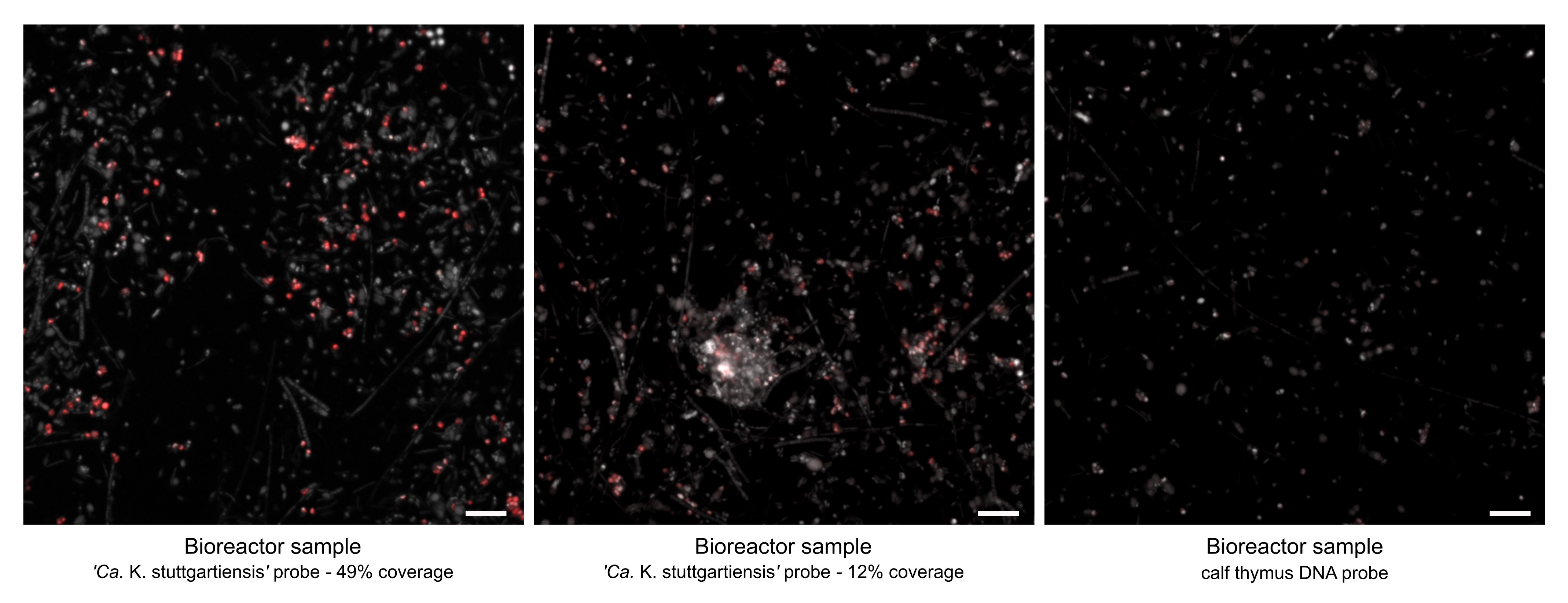

Supplement: Figure_S12_wraf138 [file figure_s12_wraf138.jpeg]

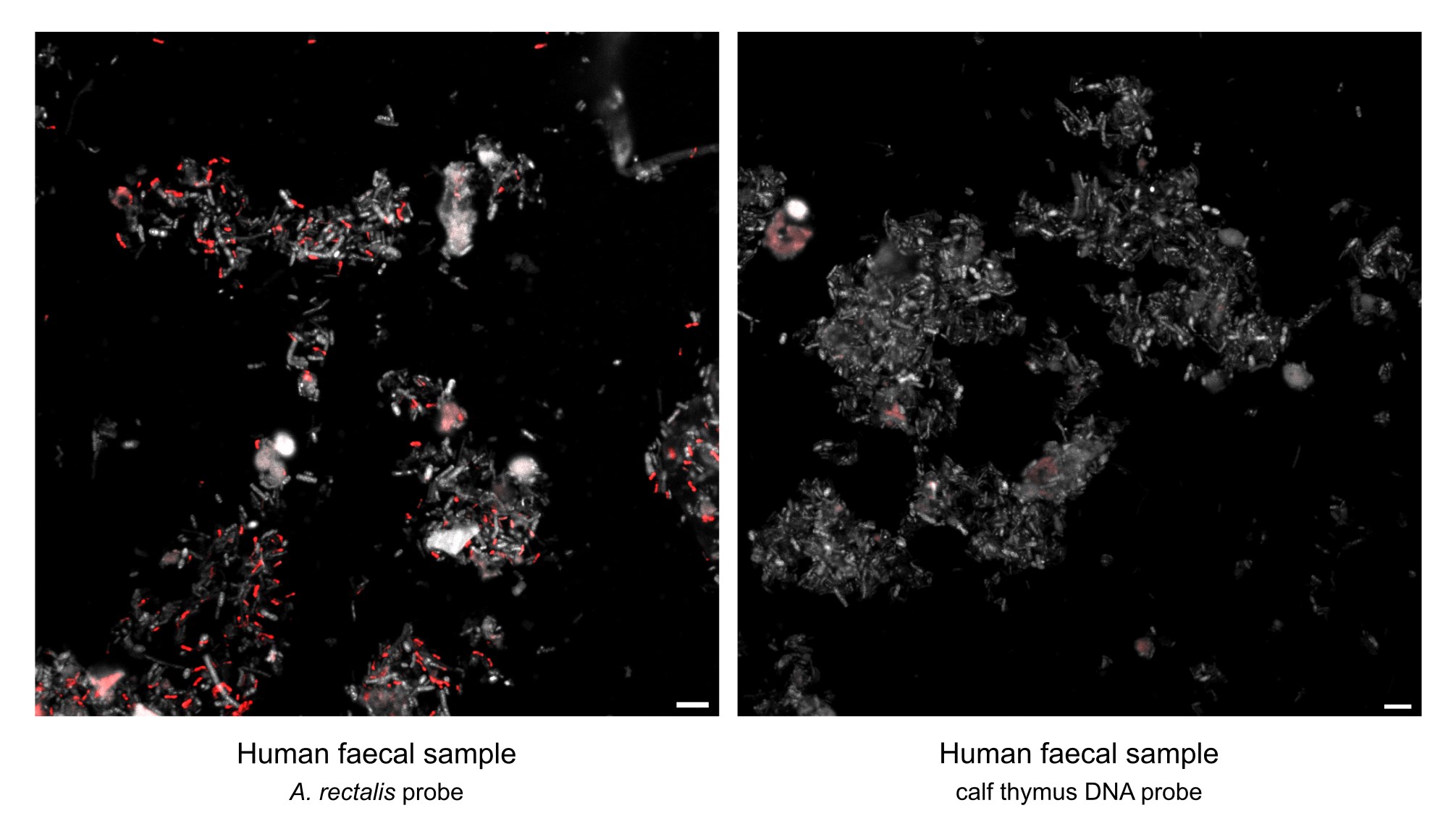

Supplement: Figure_S13_wraf138 [file figure_s13_wraf138.jpeg]

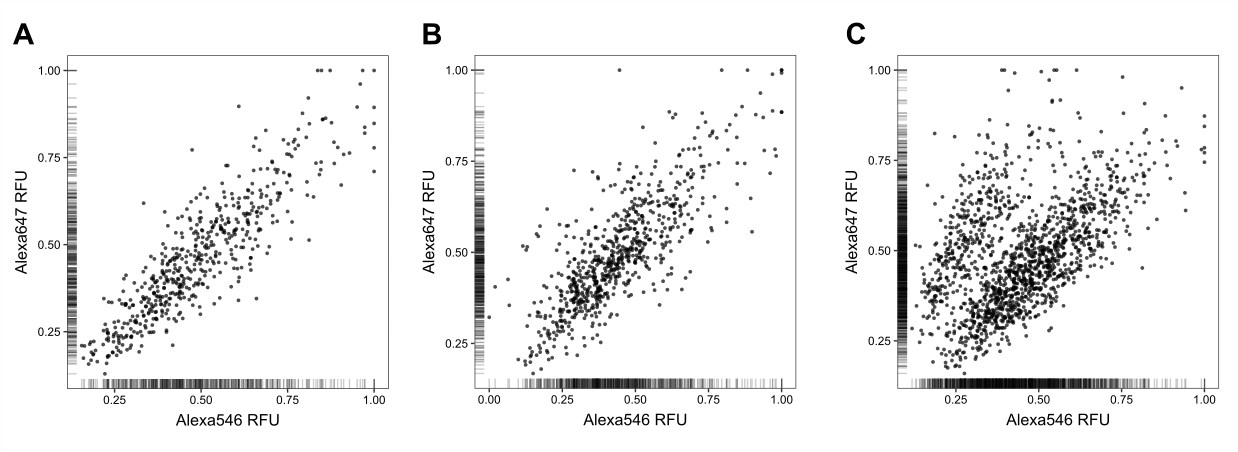

Supplement: Figure_S14_wraf138 [file figure_s14_wraf138.jpeg]
