# Supplementary material for: GenomeFISH: genome-based fluorescence in situ hybridization for strain-level visualization of microbial communities
Source: ISME J. 2025 Jul 7;19(1):wraf138. doi: 10.1093/ismejo/wraf138 (PMC12344553; doi:10.1093/ismejo/wraf138)
Supplement: Supplementary_information_wraf138 [file supplementary_information_wraf138.docx]

**GenomeFISH: genome-based fluorescence *in situ* hybridisation for strain-level visualisation of microbial communities**

**Supplementary Information**

**Supplementary Note 1**

To generate GenomeFISH polynucleotide probes, DNA of isolates, SAGs, and calf thymus (negative control) was sheared to ~130bp, a length consistent with the optimal fragment size (100bp to 500bp suggested for nucleic acid hybridisations^1^. To ensure the optimal penetration of GenomeFISH probes into cells, the length of the probes was aimed to be towards the lower optimal fragment size. Fragments were fluorescently labelled, and probe labelling efficiency was measured in nucleotide base to dye ratio (BDR, **Table S2**). For isolates, the average labelling efficiencies per 130 nucleotides were 1.6 (for Alexa546), 2.3 (Alexa594), and 2.0 fluorophores (Alexa647). Average labelling efficiencies were 1.9 (Alexa546), 2.2 (Alexa594), and 1.3 fluorophores (Alexa647) for SAGs, and 2.4, 1.8, and 2.2, respectively, for calf thymus DNA.

**Supplementary Note 2**

To generate a growth curve for *E. coli* strain EDL933, cells were grown aerobically at 37°C in LB medium (1% tryptone, 1% NaCl, and 0.5% yeast extract) in Hungate tubes in technical triplicate and biological duplicate. Optical Density was captured using the Genesys 30 Spectrophotometer (Thermo Fisher Scientific).

**Supplementary Note 3**

Two *Agathobacter rectalis* single amplified genomes (SAGs) were obtained from a single donor from samples collected 11 months apart (Nov 2022 and Oct 2023, **Table S4**). The SAG from each sample was near identical to the MAG recovered from the respective metagenome (99.78% ANI sample 1, 99.88% ANI sample 2), but the genomes differed between samples (94.8% ANI between SAGs, 98.08% ANI between MAGs), suggesting that each faecal sample contained a distinct *A. rectalis* strain.

To investigate if the two strains were unique to each sample, reads from the bulk metagenomes were aligned to each MAG (see details below). In the first metagenome, 99.4% of MAG 1-specific single nucleotide variants (SNVs) had >0 coverage (median coverage 250), while only 1.7% of MAG 2-specific SNVs had >0 coverage. This shows that the strain of the second faecal sample (Strain 2) was either absent or present only at very small abundance in the first faecal sample. The converse analysis (i.e. mapping reads from the second metagenome) yielded analogous results, showing that the strain found in the first faecal sample (Strain 1) was absent from the second faecal sample.

*Methodological details*

To find variants in the metagenomic reads, Lorikeet v0.8.2^2^ 'call' was used to call variants against the Strain 1 genome as a reference. To account for genome incompleteness in the MAGs, only variant positions in regions that aligned between the two genomes were considered. Specifically, only the coverage of those variants which could be converted between the MAGs using transanno v0.4.5 (https://github.com/informationsea/transanno) ‘liftover’ from the Strain 1 genome to the Strain 2 genome were considered. To generate the chain file required for ‘liftover’^3^, minimap2 v2.28-r1209^4^ ‘-cx asm5 --cs’ was used to align the genome, with the resulting PAF file converted to chain format using transanno ‘minimap2chain’.

After applying bcftools v1.20^5^ ‘norm -m +any’ and ‘annotate -x FORMAT/PL’ to the variant call format (VCF) file^6^ produced by Lorikeet, transanno ‘liftover’ was applied. Of the 36,854 variants, 27,807 biallelic SNVs were lifted over from the genome of Strain 2 to the genome of Strain 1.

**Supplementary Figure legends**

**Figure S1.** Representative GenomeFISH micrographs of E. coli strain EDL933 showing signal intensities when cells fixed with either 4% PFA or 1% PFA were hybridized with GenomeFISH probes generated from E. coli strain EDL933.

**Figure S2. Representative GenomeFISH micrographs for in-house cultured isolates.** GenomeFISH micrographs show *Escherichia coli* strains (CFT073, EDL933, UQ950, and C43), *Escherichia fergusonii*, *Klebsiella aerogenes*, *Pseudomonas aeruginosa*, *Megamonas funiformis*, *Bacillus subtilis*, and *Agathobacter rectalis* hybridised with their respective GenomeFISH probes. Scale bars are 5 µm.

**Figure S3.** Representative GenomeFISH micrographs of *E. coli* strain EDL933 hybridised with GenomeFISH probes generated from *E. coli* strain EDL933 (left) or calf thymus DNA (right). GenomeFISH signal appears red and DAPI signal grey. Scale bars are 5 µm.

**Figure S4.** **Comparison of signal intensities of traditional FISH and GenomeFISH performed on isolates.** Representative micrographs show signal intensities for *Escherichia coli* strain EDL933 (top panels), *Klebsiella aerogenes* (middle panels), and *Bacillus subtilis* (bottom panels). Traditional FISH probes (left panels) and GenomeFISH probes (right panels) were applied to targeted cells under respective optimal conditions and images for comparison were acquired using the same settings on the microscope for each species. Signal intensities for *E. coli* strain EDL933 when hybridised with the *E. coli* EDL933 GenomeFISH probe and *E. coli* CFT073 GenomeFISH probe (as a competitor) are also shown. Scale bars are 5 µm. Significance: *P* < 0.05 (*), *P* < 0.01 (**), and *P* < 0.001 (***).

**Figure S5.** **Representative GenomeFISH micrographs for Figure 3A.** GenomeFISH micrographs show the DAPI signal and signal intensity of probes generated from *E. coli* strain CFT073 when hybridised to four bacteria with ANI values between 74.6% and 96.6%. Scale bar is 5 um.

**Figure S6. Relationship between the GenomeFISH and DAPI signal in a target population** A) GenomeFISH micrographs showing the signal variation within a single *Escherichia coli* strain EDL933 population. The left micrograph shows the GenomeFISH signal, the right micrograph shows the DAPI signal. Scale bar is 5 µm. B) Correlation between GenomeFISH and DAPI signal intensity. Each grey dot represents a single *E. coli* EDL933 cell as seen in Panel A. C) Relationship between the GenomeFISH and DAPI signal across growth phases of *E. coli* strain EDL933. D) Growth curve showing the growth phases of *E. coli* strain EDL933.

**Figure S7**. **Image segmentation results of Figure 3C.** Signal intensity (RFU, relative fluorescence unit) of Alexa546 versus Alexa594 for each cell in a mock community consisting of *Escherichia coli* strains CFT073 and EDL933, *Escherichia fergusonii*, and *Pseudomonas aeruginosa*. Cells were hybridised with GenomeFISH probes generated from *E. coli* CFT073 in Alexa546 and from *E. coli* EDL933 in Alexa594.

**Figure S8. The effect of GC content on the hybridisation efficiency of GenomeFISH probes.** Formamide dissociation curves for four microbial species with varying GC content, including *Megamonas funiformis* (top left, 32% GC content), *Escherichia coli* strain CFT073 (top right, 50% GC), *Klebsiella aerogenes* (bottom left, 55% GC), and *Pseudomonas aeruginosa* (bottom right, 66% GC). Each microorganism was hybridised with their respective GenomeFISH probes. Normalised signal intensities are shown (GenomeFISH/DAPI signal intensities). Optimal formamide concentrations for the application of GenomeFISH on mixed microbial communities are highlighted in green.

**Figure S9. The effect of SAG coverage on the specificity and sensitivity of GenomeFISH.** Relationship between normalised GenomeFISH signal intensity (GenomeFISH/DAPI signal intensity) and single amplified genome (SAG) coverage when GenomeFISH probes generated from *Escherichia coli* strain CFT073 SAGs were hybridised to *E. coli* strains CFT073 and EDL933. A coverage of 0% represents the normalised signal intensity of cells when no probes are added to the hybridisation. Signal intensities were measured for five fields of view. Significance: *P* < 0.05 (*), *P* < 0.01 (**), and *P* < 0.001 (***).

**Figure S10.** **GenomeFISH micrographs of microbial populations in a bioreactor sample.** GenomeFISH micrographs show biomass of a bioreactor sample hybridised with GenomeFISH probes generated from ‘*Ca*. Methanoperedens nitroreducens’ (top left), ‘*Ca.* Kuenenia stuttgartiensis’ (top right), SURF-28 sp003599395 (bottom left), and ‘*Ca*. Chazhemtobacterium aquaticus’ (bottom right). GenomeFISH signal appears red and DAPI signal grey. Scale bars are 5 μm.

**Figure S11**. **Comparison of signal intensities of traditional FISH and GenomeFISH performed on a bioreactor community.** Representative micrographs show signal intensities for ‘*Ca*. Kuenenia stuttgartiensis’ (top panels) and SURF sp003599395 (bottom panels). Traditional FISH probes (left panels) and GenomeFISH probes (right panels) were applied to targeted cells under respective optimal conditions and images for comparison were acquired using the same settings on the microscope for each species. Scale bars are 5 µm. Significance: *P* < 0.05 (*), *P* < 0.01 (**), and *P* < 0.001 (***).

**Figure S12.** Representative GenomeFISH micrographs of a bioreactor community hybridised with GenomeFISH probes generated from ‘*Ca.* Kuenenia stuttgartiensis’ (49% complete SAG, left), ‘*Ca.* Kuenenia stuttgartiensis’ (12% complete SAG, middle), and calf thymus DNA (right). GenomeFISH signal appears red and DAPI signal grey. Scale bars are 5 µm.

**Figure S13.** Representative GenomeFISH micrographs of a human faecal sample hybridised with GenomeFISH probes generated from *Agathobacter rectalis* (left) or calf thymus DNA (right). GenomeFISH signal appears red and DAPI signal grey. Scale bars are 5 µm.

**Figure S14**. **Image segmentation of GenomeFISH micrographs of *A. rectalis* strains in human faecal samples.** Signal intensity (RFU, relative fluorescence unit) of cells labelled with Alexa546 versus Alexa647 for human faecal samples hybridised with GenomeFISH probes from *Agathobacter rectalis* Strain 1 (in Alexa546) and *A. rectalis* Strain 2 (in Alexa647). A and B) Signal intensities for two individual faecal samples, each containing one strain of *A. rectalis*. Plot A shows the first faecal sample that was collected and Plot B shows the second faecal sample that was collected from the same donor 11 months later. C) Signal intensities of cells in a mixture of two human faecal samples containing two *A. rectalis* strains. All samples (Plot A-C) were hybridised with GenomeFISH probes generated from both Strain 1 and 2. For each plot, signal intensities were measured for five fields of view.

**Supplementary Table legends**

**Table S1.** Details of the in-house cultured isolates and single amplified genomes (SAGs) used to optimise the GenomeFISH protocol. SAGs highlighted in green were used to test the effect of SAG coverage on signal specificity and sensitivity.

**Table S2.** Overview of GenomeFISH and FISH probes that were used in this study. The labelling efficiency of isolate and single cell DNA for the respective fluorophore the DNA was labelled with is listed for GenomeFISH probes. BDR: base to dye ratio. The formamide concentration used for the application of the traditional FISH probes is further included.

**Table S3.** Overview of the metagenome assembled genomes (MAGs), and single amplified genomes (SAGs) retrieved from the bioreactor sample, including MAG abundance and taxonomy, and fraction of the corresponding MAGs covered by the SAGs. MAGs for which SAGs were recovered are highlighted in grey. SAGs that were used to generate GenomeFISH probes are highlighted in green.

**Table S4.** Overview of the metagenome assembled genomes (MAGs), and single amplified genomes (SAGs) retrieved from two human faecal samples, including MAG abundance and taxonomy, and fraction of the corresponding MAGs covered by the SAGs. MAGs for which SAGs were recovered are highlighted in grey. SAGs that were used to generate GenomeFISH probes are highlighted in green.

**References**

1 Lichter, P. & Cremer, T. in *Human Cytogenetics: A Practical Approach* (ed D.E. Rooney and B.H. Czepulkowski) 157–192 (IRL Press, 1992).

2 Newell, R. J. P., McMaster, E. S., Craig, P., Boden, M., Tyson, G. W., Woodcroft, B. J. Lorikeet: strain-resolved metagenome analysis using local reassembly (v0.8.2). *Zenodo* (2023). <https://doi.org:https://doi.org/10.5281/zenodo.10275469>

3 Genovese, G. *et al.* BCFtools/liftover: an accurate and comprehensive tool to convert genetic variants across genome assemblies. *Bioinform* **40** (2024). <https://doi.org:10.1093/bioinformatics/btae038>

4 Li, H. Minimap2: pairwise alignment for nucleotide sequences. *Bioinform* **34**, 3094-3100 (2018). <https://doi.org:10.1093/bioinformatics/bty191>

5 Danecek, P. *et al.* Twelve years of SAMtools and BCFtools. *Gigascience* **10**, giab008 (2021).

6 Danecek, P. *et al.* The variant call format and VCFtools. *Bioinform* **27**, 2156-2158 (2011). <https://doi.org:10.1093/bioinformatics/btr330>
