# Supplementary material for: GenomeFISH: genome-based fluorescence in situ hybridization for strain-level visualization of microbial communities
Source: ISME J. 2025 Jul 7;19(1):wraf138. doi: 10.1093/ismejo/wraf138 (PMC12344553; doi:10.1093/ismejo/wraf138)
Supplement: Figure_S5_wraf138 [file figure_s5_wraf138.pdf]

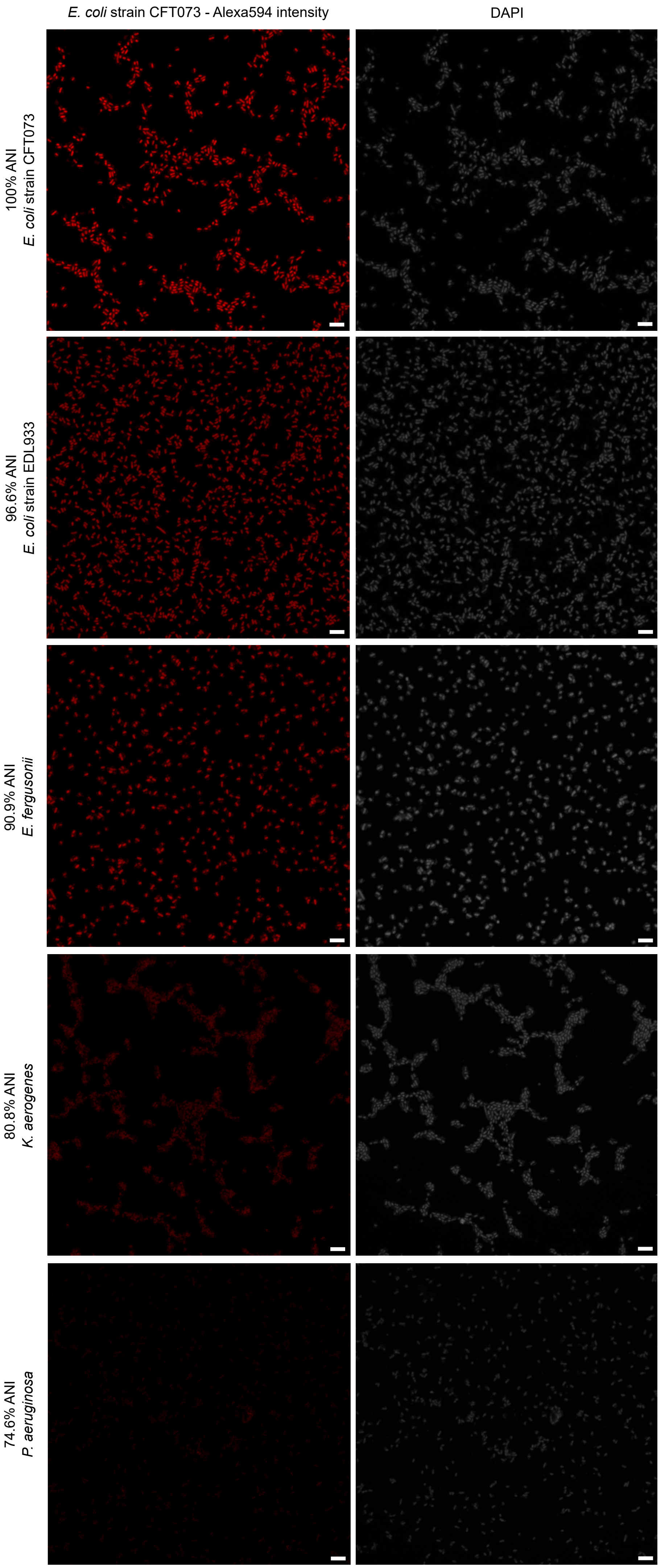

E. coli strain CFT073 - Alexa594 intensity
DAPI
100% ANI
E. coli strain CFT073
96.6% ANI
E. coli strain EDL933
90.9% ANI
E. fergusonii
80.8% ANI
K. aerogenes
74.6% ANI
P. aeruginosa
